# Quaking Is a Key Regulator of Endothelial Cell Differentiation, Neovascularization, and Angiogenesis

**DOI:** 10.1002/stem.2594

**Published:** 2017-03-05

**Authors:** Amy Cochrane, Sophia Kelaini, Marianna Tsifaki, James Bojdo, Marta Vilà‐González, Daiana Drehmer, Rachel Caines, Corey Magee, Magdalini Eleftheriadou, Yanhua Hu, David Grieve, Alan W. Stitt, Lingfang Zeng, Qingbo Xu, Andriana Margariti

**Affiliations:** ^1^ The Wellcome‐Wolfson Building, Centre for Experimental Medicine, Queen's University BelfastUnited Kingdom; ^2^ Cardiovascular DivisionKing's College LondonLondonUnited Kingdom

**Keywords:** Induced pluripotent stem cells, Endothelial cell differentiation, Vascular disease, Angiogenesis, QKI‐5

## Abstract

The capability to derive endothelial cell (ECs) from induced pluripotent stem cells (iPSCs) holds huge therapeutic potential for cardiovascular disease. This study elucidates the precise role of the RNA‐binding protein Quaking isoform 5 (QKI‐5) during EC differentiation from both mouse and human iPSCs (hiPSCs) and dissects how RNA‐binding proteins can improve differentiation efficiency toward cell therapy for important vascular diseases. iPSCs represent an attractive cellular approach for regenerative medicine today as they can be used to generate patient‐specific therapeutic cells toward autologous cell therapy. In this study, using the model of iPSCs differentiation toward ECs, the QKI‐5 was found to be an important regulator of STAT3 stabilization and vascular endothelial growth factor receptor 2 (VEGFR2) activation during the EC differentiation process. QKI‐5 was induced during EC differentiation, resulting in stabilization of STAT3 expression and modulation of VEGFR2 transcriptional activation as well as VEGF secretion through direct binding to the 3′ UTR of STAT3. Importantly, mouse iPS‐ECs overexpressing QKI‐5 significantly improved angiogenesis and neovascularization and blood flow recovery in experimental hind limb ischemia. Notably, hiPSCs overexpressing QKI‐5, induced angiogenesis on Matrigel plug assays in vivo only 7 days after subcutaneous injection in SCID mice. These results highlight a clear functional benefit of QKI‐5 in neovascularization, blood flow recovery, and angiogenesis. Thus, they provide support to the growing consensus that elucidation of the molecular mechanisms underlying EC differentiation will ultimately advance stem cell regenerative therapy and eventually make the treatment of cardiovascular disease a reality. The RNA binding protein QKI‐5 is induced during EC differentiation from iPSCs. RNA binding protein QKI‐5 was induced during EC differentiation in parallel with the EC marker CD144. Immunofluorescence staining showing that QKI‐5 is localized in the nucleus and stained in parallel with CD144 in differentiated ECs (scale bar = 50 µm). stem cells *2017* Stem Cells *2017;35:952–966*

## Introduction

Cardiovascular disease is a leading cause of mortality worldwide with pathology being significantly driven by progressive vascular endothelial cell (EC) dysfunction which regulates key pathogenic events such as infiltration of inflammatory cells, vascular smooth muscle proliferation, and platelet aggregation 1, 2. As cardiovascular disease progresses, ECs become depleted from the vascular lumen, and, without adequate replacement, non‐perfusion and tissue ischaemia ensues. Repair and regeneration of ECs has been an important research focus for many years. As an important therapeutic avenue, the delivery of adult progenitor or stem cells to repair damaged vasculature has faced many limitations, such as identification and availability of appropriate, efficacious cell‐types for therapy 3. However, recently, the ability to derive ECs from induced pluripotent stem cells (iPSCs) has extended the scientific scope for regenerative medicine 4, 5, 7.

Cell reprogramming is offering new avenues for regenerative medicine with iPSCs proving able to differentiate into nearly all types of cells within the body 6, 7, 8. This is a unique characteristic of iPSC technology, which offers a significant potential for cell‐based therapies toward repairing tissues or organs destroyed by injury, degenerative diseases, aging, or cancer 4, 5, 8, 9. Generation of iPSCs also offers a promising strategy to create patient‐specific cells [7, 10–13] with building evidence that these cells have therapeutic efficacy in animal models of disease [3, 5, 14–16]. The concept of cell reprogramming is powerful 17, 18, 19, 20 and has, so far, allowed the development of dynamic approaches to generate functional cells of interest and to switch cell fate 20. We and others have recently demonstrated novel strategies of direct reprogramming toward functional ECs 21, 22. Both iPSCs and directly reprogrammed cells (partial iPSC–PiPSCs) can serve as useful tools not only for derivation of functional cells but also for establishing efficient protocols of differentiation and developing platforms to investigate the underlying mechanisms.

Vascular cell differentiation is achieved through the coordination of diverse molecular pathways 21, 23, 24, 25, 26, 27, 28. Due to this, there is a need to better understand the complex dynamics behind RNA regulation and how this, in turn, influences transcriptional control and protein expression. Elucidation of these complex molecular signals, which are evoked during iPSC and PiPSC differentiation toward ECs, may allow specific targeting of their activities to enhance cell differentiation and promote tissue regeneration. In an effort to elucidate the imperative mechanisms of RNA regulation during EC differentiation from iPSCs, this study focused on the RNA‐binding protein Quaking (QKI). QKI is a member of the “STAR” (signal transduction and activation of RNA) family of proteins. These proteins are characterized by the presence of at least one RNA‐binding motif (QKI containing a KH domain), SH2 and SH3 domains and potential phosphorylation sites. This suggests their involvement in signal transduction pathways 29, 30, pre‐mRNA splicing 31, exportation of mRNAs from the nucleus 32, protein translation, and mRNA stability. Functional studies of the mouse QKI gene have revealed that it has a key function in embryonic blood vessel formation and remodeling 33, 34, 35, 37. During vascular cell differentiation and reprogramming processes, QKI could play an important regulatory role in gene expression at the post‐transcriptional level in either a miRNA‐dependent or independent manner. Alternative splicing of genes, a process that RNA binding proteins are highly involved in, generates many forms of mature mRNA from the same gene is an essential process in development and a potent way to regulate gene expression at a post‐transcriptional level. QKI has a number of alternative splicing isoforms, three of which have been associated with vascular development; QKI‐5, QKI‐6, and QKI‐7. This further reinforces the importance of QKI in protein regulation and development. Each isoform contains identical RNA binding domains and differs only by its carboxy‐terminal ends 36, 37. In this study, we focus on the QKI splicing isoform 5 (QKI‐5). QKI‐5 is most highly expressed during EC differentiation from iPSCs, it is an essential player during embryogenesis and notably, QKI‐5 null mice are embryonic lethal 38, 39, 41.

We provide strong evidence that QKI‐5 plays a critical role in the differentiation of ECs derived from iPSCs and acts as a key regulator of CD144 stabilization and vascular endothelial growth factor receptor 2 (VEGFR2) activation through STAT3 signaling. The RNA binding protein QKI‐5 directly binds to 3′ UTR of STAT3 and induced STAT3 mRNA stabilization. Remarkably, QKI‐5 improved neovascularization and blood flow recovery in a hind limb ischemic model. Notably, human iPSCs (hiPSCs) overexpressing QKI‐5 (OE‐QKI‐5) induced angiogenesis in Matrigel plug assays in vivo only 7 days after subcutaneous injection in SCID mice, highlighting, thus, a pivotal role of QKI‐5 in neovascularization, blood flow recovery and angiogenesis.

## Materials and Methods

Cell culture media, serum, and cell culture supplements were purchased from ATCC, England, UK, https://www.lgcstandards-atcc.org/?geo_country=gb Millipore, LONZA; England, UK, http://www.lonza.com/ and Thermo Fisher Scientific; England, UK, http://www.thermofisher.com/uk/en/home.html. Antibodies against VE‐cadherin (CD144) (ab33168), PECAM (CD31) (ab28364), Flk‐1 (ab9530), STAT3 (ab119352), pSTAT3 (ab76315), JAK‐1 (ab47435), GAPDH (ab8245), OCT4 (ab19857), eNOS (ab66127), and QKI (ab126742) were purchased from Abcam. Antibodies against vWF (SC‐8068) and QKI (SC‐103851) were purchased from Santa Cruz; Heidelberg, Germany, https://www.scbt.com/scbt/home;jsessionid=jQ9cedHXnegG6a6cJZbpXPfwu6Z4K8PRhaZidcYh8LrI_ccsHWgp!‐97074204#. Antibodies against QKI‐5 (AB 9904) were purchased from Millipore; England, UK, http://www.merckmillipore.com/GB/en?ReferrerURL=http%3A%2F%2Fwww.bing.com%2Fsearch%3Fq%3Dmillipore%26src%3DIE‐TopResult%26FORM%3DIETR02%26conversationid%3D.

### Mouse iPSC and Embryonic Stem Cell Culture and Differentiation

Mouse iPSCs were generated in our laboratory using a similar approach as described previously 20, 40. Mouse iPSCs were cultured in gelatin‐coated flasks (phosphate buffer saline [PBS] containing 0.02% gelatin from bovine skin; Sigma‐Aldrich, SIGMA; England, UK http://www.sigmaaldrich.com/united-kingdom.html) in Dulbecco's modified Eagle's medium (ATCC) supplemented with 10% fetal bovine serum (FBS), 100 IU/ml penicillin, and 100 μg/ml streptomycin (Thermo Fisher Scientific), 10 ng/ml recombinant human leukemia inhibitory factor (Millipore); and 0.1 mM 2‐mercaptoethanol (Invitrogen) in a humidified incubator supplemented with 5% CO_2_. The cells were passaged every 2 days at a ratio of 1:6. Differentiation of iPSCs was induced by seeding the cells on type IV mouse collagen (5 μg/ml)‐coated dishes in differentiation media (DM) containing α‐modified Eagle's medium supplemented with 10% FBS (Invitrogen), 0.05 mM 2‐mercaptoethanol, 100 units/ml penicillin, and 100 μg/ml streptomycin in the presence of 25 ng/ml VEGF for the time points indicated.

### hiPSCs Differentiation and Human Umbilical Vein ECs

hiPSCs were pre‐differentiated in low attachment plates using StemPro serum free media supplemented with Bone Morphogenetic Protein 4 (BMP4), Activin A, fibroblast growth factor (FGF), and VEGF for 5 days. The pre‐differentiated cells were seeded on fibronectin (Sigma‐Aldrich), while KDR endothelial precursor cells were magnetically sorted on day 6 using MicroBeads Kit (Miltenyi BIotec) and culturing in EGM‐2 media (LONZA) for 3–9 days. QKI‐5 was overexpressed or knockdown by lentiviral gene transfer on day 3 after KDR selection and the cells were harvested on day 3 and subjected to further analysis or labeled for the in vivo Matrigel plug assays. Human umbilical vein ECs (HUVECs) were seeded on collagen I coated plates and cultured in EGM‐2 media. QKI‐5 was overexpressed by lentiviral gene transfer and subjected to further analysis.

### Experimental Hind Limb Ischemia

The mouse hind limb ischemia model was performed as described previously 21, 42. iPS‐ECs were infected with FUW‐QKI‐5 or control empty vectors on day 4 of EC differentiation. After 48 hours, the cells were trypsinized and labeled with Molecular Probes Vybrant Cell Labeling (MP22885) before being injected intramuscularly into the adductors of C57BL**/**6 wild type mice.

### Statistical Analysis

Data are expressed as the mean ± SEM and were analyzed using GraphPad Prism 5 software with a two‐tailed Student's *t* test for two groups or pairwise comparisons or analysis of variance (ANOVA). A value of *, *p* < .05; *, *p* < .05; **, *p* < .01; ***, *p* < .001 was considered significant.

Detailed Methods and Materials are in Supporting Information Appendix, Experimental Procedures

## Results

### Pluripotent Stem Cells Differentiation toward ECs

**Figure 1:**
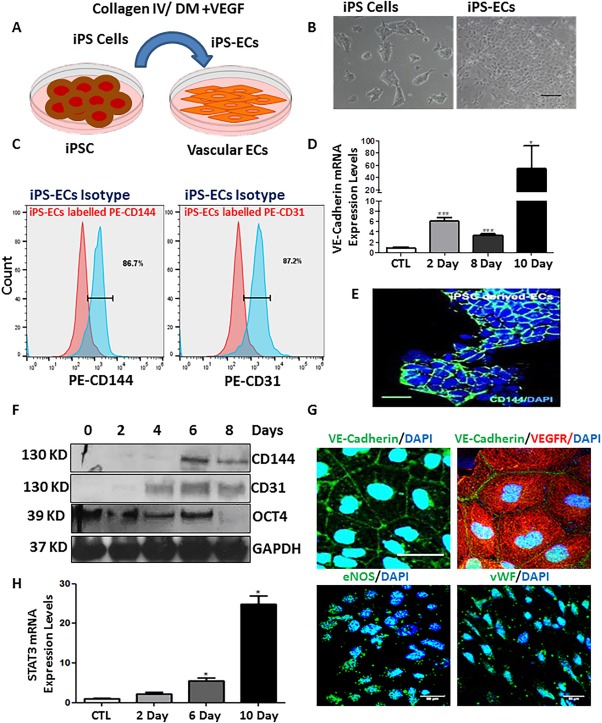
Induced pluripotent stem cell differentiation toward endothelial cells (ECs). Mouse induced pluripotent stem cells (iPSCs) were cultured on 0.02% gelatin with media supplemented with leukaemia inhibitory factor (LIF) to maintain pluripotency. To induce EC differentiation iPSCs were seeded onto plates coated with collagen IV and differentiation media supplemented with 25 ng/ml vascular endothelial growth factor (DM + V). **(A)**: Schematic representation showing differentiation process. **(B)**: Images show morphology of iPSCs (left panel) and of their differentiated EC counterparts (right panel) scale bar = 50 μm. **(C)**: FACs analysis showing expression of CD144 (left panel) and CD31 (right panel) of ECs derived from iPSCs. **(D, F)**: EC marker expression increased in a time‐dependent manner during differentiation at both mRNA and protein level, while the pluripotent marker OCT4 decreased during EC differentiation in a time‐dependent manner (data are mean ± SEM (*n* = 3), *, *p* < .05; ***, *p* < .001). **(E)**: Immunofluorescence confocal image showing that the 6 days differentiated ECs express the EC specific marker CD144, scale bar = 50 μm. **(G)**: iPS‐ECs expressed EC markers CD144, FLK‐1, eNOS, and VWF as shown by confocal immunofluorescent images, scale bar = 50 μm. **(H)**: The transcription factor STAT3 was progressively expressed during EC differentiation (data are mean ± SEM [*n* = 3], *, *p* < .05). Abbreviations: DAPI, 4′,6‐Diamidine‐2′‐phenylindole dihydrochloride; DM, differentiation medium; EC, endothelial cell; iPSC, induced pluripotent stem cell; VEGF, vascular endothelial growth factor.

Mouse iPSCs were differentiated toward ECs by seeding on collagen IV and DM supplemented with 25 ng/ml VEGF (DM + V) for 2–10 days. The differentiated ECs adopted a typical EC morphology (Fig. 1A, 1B). The efficiency of EC differentiation from mouse iPSCs was very high as FACs analysis is shown in Figure 1C (86.7% positive cells for CD144 [VE‐Cadherin] and 87.2% positive cells for CD31 were obtained). These results were confirmed in both mRNA (Fig. 1D) and protein levels (Fig. 1E, 1F). During EC differentiation the pluripotent marker OCT4 was suppressed allowing the expression of the EC specific markers CD144 and CD31 to occur in a time point dependent manner (Fig. 1F). Indeed, immunofluorescence confocal microscope images revealed that ECs derived from iPSCs expressed EC markers (CD144, FLK‐1, eNOS, and vWF) which are the characteristics of ECs (Fig. 1G). We also observed that the expression of the transcription factor STAT3 is a known essential player in VEGF signaling during vascular development. We are reporting that STAT3 was progressively induced during EC differentiation (Fig. 1H). Taken together, these data clearly demonstrate that mouse iPSCs have been differentiated toward ECs based on a highly efficient approach.

### RNA‐Binding Protein QKI Is Induced during EC Differentiation from iPSCs

**Figure 2:**
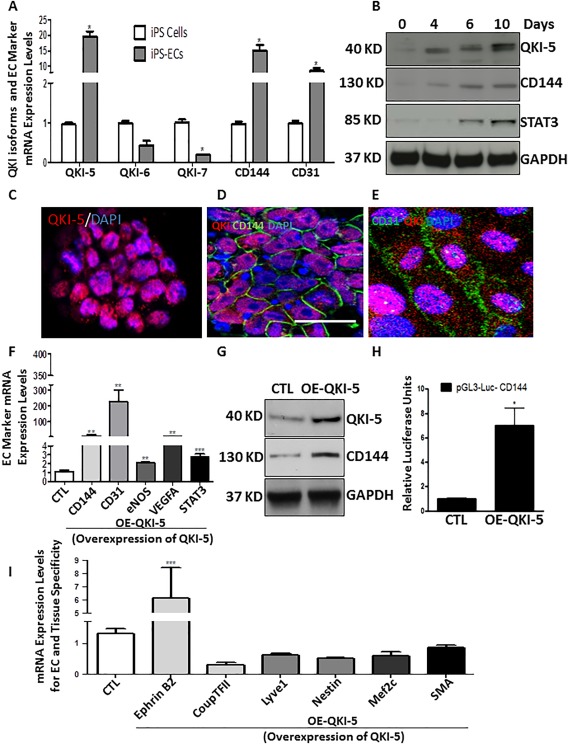
The RNA binding protein QKI‐5 is induced during endothelial cell (EC) differentiation from induced pluripotent stem cells (iPSCs). **(A)**: RNA binding protein QKI‐5 was induced during EC differentiation in parallel with the EC markers CD144 and CD31.The expression levels of QKI‐6 and QKI‐7 were not changed or decreased, respectively. **(B)**: Western blots showing that QKI‐5 protein was progressively expressed in parallel with the EC marker CD144 and the transcription factor STAT3 in time point experiments from 0 to 10 days during EC differentiation. **(C)**: Immunofluorescence staining showing that QKI‐5 is localized in the nucleus and in parallel with CD144 in differentiated ECs **(D)** (scale bar = 50 µm) and **(E)** with CD31 in mature ECs (human umbilical vein ECs), scale bar = 25 µm. **(F)**: iPS‐ECs were infected or transfected on day 4 with QKI‐5 or an empty vector. After 48 hours, QKI‐5 overexpression induced mRNA expression of endothelial markers CD144, CD31, eNOS, VEGFA, and the transcription factor STAT3. **(G)**: QKI‐5 induced protein expression of CD144. **(H)**: iPS‐ECs were differentiated for 3 days and transfections with the reporter of CD144 (VE‐cadherin) construct (pGL3‐Luc‐CD144) in the presence or absence of QKI‐5 were performed. Cells were harvested on day 5 of EC differentiation when luciferase assays demonstrated that QKI‐5 induced transcriptional activation of the VE‐cadherin promoter. **(I)**: iPS‐ECs were differentiated for 4 days and transfected with QKI‐5 or an empty vector. After 48 hours, QKI‐5 overexpression directed differentiation toward arterial ECs (Ephrin B2) specifically and not toward venous (CoupTFII) or lymphatic (Lyve1) ECs or other tissues such as nerve (Nestin), cardiac (Mef2c) or smooth muscle cell (SMA) (data are means ± SEM [*n* = 3]; *, *p* < .05; **, *p* < .01; ***, *p* < .001). Abbreviations: CTL, control; EC, endothelial cell; iPSC, induced pluripotent stem cell.

QKI‐5 was significantly induced during EC differentiation from iPSCs and this occurred in parallel with the EC markers CD144 and CD31 (Fig. 2A). It is important to note that only QKI‐5 mRNA splicing isoform was induced during EC differentiation. QKI‐6 and QKI‐7 expression levels were also tested showing no change or decrease, respectively (Fig. 2A). Time‐course experiments also demonstrated that QKI‐5 was progressively induced after 4 days post‐differentiation from iPSCs (Fig. 2B). There was a comparable induction of CD144 and STAT3 (Fig. 2B). Immunofluorescence images demonstrated that the alternative splicing isoform QKI‐5 was localized to the EC nucleus in both mouse iPSC‐derived ECs and in HUVECs (Fig. 2C–2E). Also in both EC‐types, CD144 and CD31 were expressed in the plasma membrane (Fig. 2D, 2E). These results demonstrated that QKI‐5 expression is induced during EC differentiation from mouse iPSCs, and its expression is maintained in mature ECs.

A number of additional experiments were performed to determine whether QKI‐5 plays a regulatory role in iPSC differentiation toward ECs. Initially, a pure population (100%) of differentiated ECs was obtained based on CD144 selection, as it is a robust EC‐specific marker. Based on this pure population of ECs derived from iPSCs, a high expression of QKI‐5 was observed and this was comparable to the EC markers CD144, VEGFA, and the transcription factor STAT3 (Supporting Information Fig. S1A–S1F).

Further experiments performed on mouse embryonic stem cells (ESCs) differentiated to ECs, using a similar approach as the iPSCs, verified that QKI‐5 expression was induced in parallel with CD144 expression (Supporting Information Fig. S2A–S2D). These results indicated that QKI‐5 may be implicated in differentiation of ECs derived from mouse iPSC and ESCs. To elucidate the underlying mechanisms regulated by QKI‐5 in EC differentiation, QKI‐5 was overexpressed by lentiviral gene transfer at day 4 of EC differentiation using ECs derived from iPSCs. The cells were harvested on day 6 and subjected to further analysis. QKI‐5 overexpression significantly enhanced expression of the endothelial markers CD144, CD31, eNOS, and VEGFA and the transcription factor STAT3 (Fig. 2F, 2G). Surprisingly, QKI‐5 overexpression also induced the in activation of the CD144 (VE‐Cadherin) promoter as revealed by luciferase assays (Fig. 2H).

Importantly, QKI‐5 also induced the expression of the arterial specific EC marker Ephrin B2, but it did not affect the expression of venous (CoupTFII) and lymphatic (Lyve1) markers. Moreover, QKI‐5 induced EC differentiation in an EC‐specific manner, while markers of other cells lineages such as neuronal (Nestin), cardiac (Mef2c), or smooth muscle cell (SMA) were not altered by QKI‐5 overexpression (Fig. 2I). These data reveal that QKI‐5 induced EC differentiation toward an arterial lineage.

### QKI‐5 Is Capable of Initiating EC Differentiation from iPSCs

Further experiments have revealed that QKI‐5 leads to the stabilization of VE‐Cadherin adhesion molecule during the EC differentiation process. When differentiated ECs were treated with Actinomycin D for 6 hours or cyclohexamide for 24 hours, CD144 (VE‐Cadherin) expression was stabilized in the presence of QKI‐5 (Supporting Information Fig. S3A–S3D), respectively. Moreover, when differentiated ECs were treated with Actinomycin D in a time point experiment from 0 to 24 hours, CD144 expression was stabilized as a decay curve as is shown in Supporting Information Figure S3E. These results demonstrate that QKI‐5 induced the expression and stabilization of CD144 during EC differentiation. Additional experiments have been conducted to address the important question whether QKI‐5 is able to initiate EC differentiation from iPSCs. When QKI‐5 was overexpressed in undifferentiated iPSCs, the cells started expressing the arterial marker Ephrin B2 3 days later, while venous, lymphatic, or markers of other cell lineages, such as Nestin and SMA, were not expressed (Supporting Information Fig. S4A). Importantly, EC markers were highly induced upon QKI‐5 overexpression in early stages of EC differentiation (Supporting Information Fig. S4B). Finally, to shed light on the upstream signaling, which activates QKI‐5 expression in early stages of EC differentiation from iPSCs, transcription factor binding analysis on the QKI‐5 promoter has predicted a potential binding site for the transcription factor ETS1. ETS factors, and in particular ETS1 and Etv2, have been reported as key factors that regulate endothelial development 43. Interestingly, ETS1 is induced during EC differentiation from iPSCs in a time‐dependent manner (Supporting Information Fig. S4C), while overexpression of ETS1 in early stages of EC differentiation has led to induction of QKI‐5 (Supporting Information Fig. S4D). These results demonstrate that QKI‐5 is able to initiate EC differentiation from iPSCs, and ETS1 may be implicated in the activation of QKI‐5 in the early stages of EC differentiation.

### QKI‐5 Induced VEGFR2 Activation and VEGF Secretion through Direct Binding of the 3′UTR Region of STAT3

**Figure 3:**
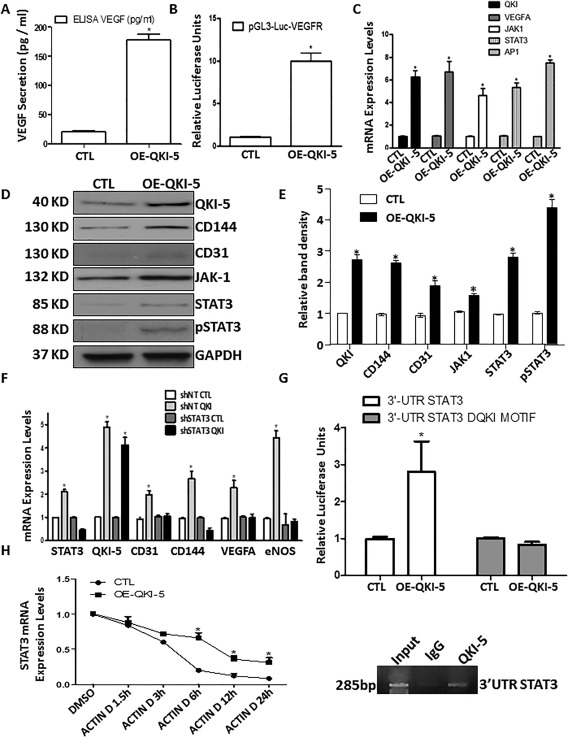
QKI‐5 induced vascular endothelial growth factor receptor 2 (VEGFR2) activation and VEGF secretion through direct binding of the 3′UTR region of STAT3. Overexpression of QKI‐5 by lentiviral gene transfer induced the secretion of VEGF on day 6 of the endothelial cell (EC) differentiation process **(A)** and the transcriptional activation of the VEGFR2 **(B)**. **(C)**: Real time polymerase chain reaction (PCR) data showing that QKI‐5 leads to activation of VEGFA, JAK‐1, STAT3, and AP1. **(D)**: Western blots showing that overexpression of QKI‐5 induced the expression of EC markers CD144, and CD31 in parallel to induction of JAK‐1, STAT3, and phosphorylation of STAT3 (quantification in E). **(F)**: STAT3 was knocked down by shRNA on day 3 of EC differentiation, and QKI‐5 was overexpressed next day. Real data PCR data reveal that STAT3 knocked down ablated activation of EC markers CD31, CD144, eNOS, and VEGFA mediated by QKI‐5. The cells were harvested on day 6 of EC differentiation. **(G)**: Luciferase assays have shown that QKI‐5 activated the 3′UTR of STAT3. QKI‐5 was unable to activate the 3′UTR of STAT3 when the QKI motif was deleted. (G, lower panel) RNA binding assays have confirmed that QKI binds directly to the 3′UTR of STAT3. **(H)**: When differentiated ECs were treated with Actinomycin D in a time point experiment from 0 to 24 hours STAT3 expression was stabilized as a decay curve is shown (data are means ± SEM [*n* = 3]; *, *p* < .05). Abbreviations: CTL, control; VEGF, vascular endothelial growth factor.

Formation of vascular networks is highly dependent on VEGF as a critical regulator of EC differentiation and vasculogenesis during development. Overexpression of QKI‐5 by lentiviral gene transfer induced the secretion of VEGF on day 6 during the EC differentiation process (Fig. 3A). Similarly, QKI‐5 overexpression led to activation of VEGFR2 during EC differentiation from iPSCs (Fig. 3B). To shed light on the signaling cascade initiated by the VEGFR transcriptional activation, the expression of the transcription factors STAT3, JAK‐1, and AP‐1 was assessed. Over‐expression of QKI‐5 leads to enhanced transcriptional activation of the VEGFR2 signaling pathway, which seems to be related to increased expression of VEGFA and autocrine stimulation of its cognate receptor (Fig. 3C). Notably, overexpression of QKI‐5 induced the expression of EC markers, CD144 and CD31, in parallel with the induction of JAK‐1, STAT3, and phosphorylation of STAT3 (Fig. 3D, and quantification in Fig. 3E). These results revealed that QKI‐5 is implicated in the activation of the VEGFR2‐regulatory binding sites AP1, STAT3, as well as STAT3 phosphorylation. Additional experiments were performed to investigate the nature of QKI‐5‐mediated activation of VEGFR. When STAT3 was knocked down by shRNA during EC differentiation, the previously observed upregulation of EC markers (CD144, CD31, and eNOS) and VEGFA, mediated by QKI‐5 was ablated (Fig. 3F). Furthermore, luciferase assays revealed that knockdown of STAT3 by shRNA ablated the QKI‐5‐mediated activation of VEGFR2 (Supporting Information Fig. S5A). In agreement with the above results, when the differentiated ECs were treated with inhibitors to block the JAK‐1 and STAT3 pathways, QKI‐5 did not activate the EC markers CD31, CD144, eNOS, FLK‐1 (VEGFR2), and VEGFA (Supporting Information Fig. S5B). Taken together, these data demonstrate that QKI‐5 modulates the activation of VEGFR and EC differentiation through the transcription factor STAT3. Interestingly, the KH DOMAIN‐mutant QKI‐5 constructs did not induce the expression of the EC markers and STAT3 signaling when it was overexpressed in the iPSCs (Supporting Information Fig. S6A–S6D). These results suggest that QKI‐5 binds candidate RNAs in the JAK1‐STAT3 signaling pathway and regulates EC differentiation. Importantly, luciferase assays have shown that QKI‐5 activated the 3′UTR of STAT3. Remarkably, QKI‐5 was unable to activate the 3′UTR of STAT3 when the QKI motif was deleted (Fig. 3G). Moreover, RNA binding assays have confirmed that QKI binds directly to the 3′UTR of STAT3 (Fig. 3G, lower panel). Moreover, when differentiated ECs were treated with Actinomycin D in a time point experiment from 0 to 24 hours, STAT3 expression was stabilized as a decay curve is shown in Figure 3H. These results clearly demonstrate that the RNA binding protein QKI‐5 induced VEGFR2 activation and VEGF secretion through direct binding of the 3′UTR of STAT3.

### QKI‐5 Has a Critical Role during EC Differentiation and Vascular Tube Formation

**Figure 4:**
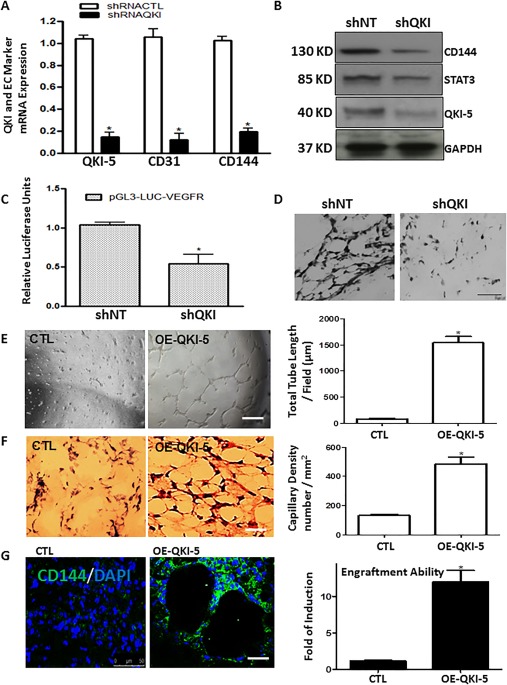
QKI‐5 plays a critical role during endothelial cell (EC) differentiation and vascular tube formation both in vitro and in vivo. QKI‐5 knockdown by shRNA in iPS‐ECs at day 4 of differentiation resulted in suppression of **(A)** mRNA expression of the EC markers CD144 and CD31 (data are means ± SEM [*n* = 3]; *, *p* < .05), and **(B)** protein expression of CD144 and the transcription factor STAT3, when assessed on day 6. **(C)**: shRNA knockdown of QKI‐5 resulted in suppression of the transcriptional activity of the vascular endothelial growth factor receptor 2 (VEGFR2) during EC differentiation, as shown by luciferase assays. Nontargeting (NT) control for knockdown experiments using shRNA. These experiments were performed on day 3 and analysed on day 5 of EC differentiation (data are means ± SEM [*n* = 3]; *, *p* < .05). **(D)**: shRNA knockdown of QKI‐5 during EC differentiation suppressed the formation of vascular‐like tubes in vivo in Matrigel plug assays. iPS‐ECs were infected with QKI‐5 shRNA on day 4 of EC differentiation, and 48 hours later, mixed with Matrigel and subcutaneous injected in to mice, prior to analysis of Matrigel plugs 7 days later; scale bar = 50 µm. Induced pluripotent stem cells were seeded on collagen IV‐coated plates and cultured in differentiated media in the absence of VEGF for 4 days when QKI‐5 or control plasmids were introduced by lentiviral gene transfer. Two days later, cells were mixed with Matrigel for in vitro plug assays. **(E)**: QKI‐5 formed vascular structures within a few hours in vitro in comparison with the control where less defined vascular structures were observed (e, left panel, with right panel showing quantification as total tube length/field (µm) (data are means ± SEM [*n* = 3]; *, *p* < .05). **(F)**: Similarly, cells were subjected to Matrigel plug assays in vivo which showed that QKI‐5‐expressing cells form well‐defined vascular structures at 7 days in comparison with the control where less well‐formed vascular structures were observed (**(F)** left panel, with quantification of capillary density [number per square millimeter] in the right panel) (data are means ± SEM [*n* = 3]; *, *p* < .05). **(G)**: Frozen sections from the in vivo Matrigel plugs were stained for CD144 to demonstrate that overexpression of QKI‐5 induced the formation of vascular structures and enhanced engraftment ability compared with the control cells (data are means ± SEM [*n* = 3]; *, *p* < .05). Scale bar = 50 µm. Abbreviations: CTL, control; EC, endothelial cell.

When QKI‐5 expression was suppressed by shRNA during EC differentiation, this resulted in a significant suppression of the EC markers CD144 and CD31 (Fig. 4A, 4B) and the transcription factor STAT3 (Fig. 4B). Moreover, knockdown of QKI‐5 caused inhibition in the transcriptional activation of VEGFR (Fig. 4C). Furthermore, knockdown of QKI‐5 by shRNA resulted in inhibition of angiogenesis in Matrigel plugs in vivo (Fig. 4D), which further reinforced the notion that QKI‐5 plays a critical role during EC differentiation and subsequent angiogenic function of the derived cells.

### QKI‐5 Induced Angiogenesis in Both in Vitro and in Vivo

QKI‐5 appears to play an important role in iPSC‐derived ECs during the formation of vascular networks in vitro and in vivo. These processes are greatly dependent on the presence of VEGF, although these experiments were conducted in the absence of exogenous VEGF. Notably, overexpression of QKI‐5 by lentiviral gene transfer induced vascular tube formation in vitro (Fig. 4E, quantified in the right panel as total tube length/field [µM]) and vessel formation in vivo (Fig. 4F, quantified in the right panel as capillary density) when compared with controls. CD144 immunofluorescence staining verified the presence of differentiated cells in in vivo vascular tubes and demonstrated that QKI‐5 over‐expressing iPSCs displayed well‐formed vascular structures and enhanced engraftment ability when compared with the control cells (Fig. 4G). Moreover, the nascent vessels in vivo (matrigel plugs) were stabilized by pericytes/vascular smooth muscle cells in the presence of QKI‐5 as smooth muscle alpha‐actin (SMA) staining is shown in Supporting Information Figure S7. These results demonstrate that QKI‐5 induced the angiogenesis in both in vitro and in vivo Matrigel plug assays.

### QKI‐5 Significantly Improved Neovascularization and Blood Flow Recovery in the Hind Limb Ischemic Model

**Figure 5:**
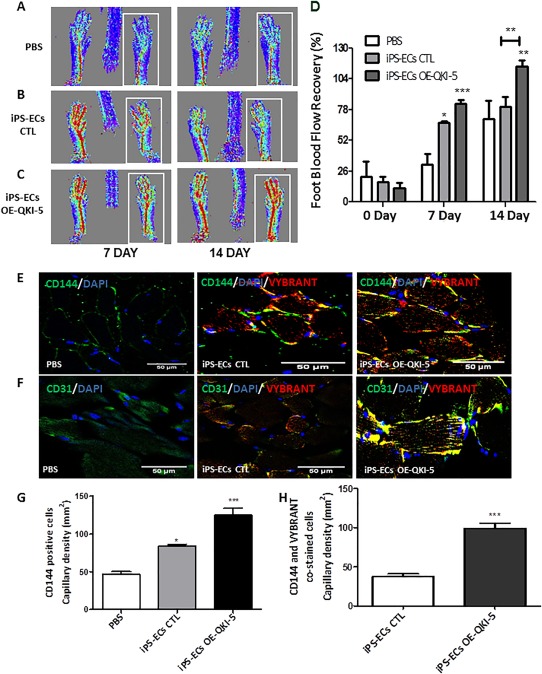
QKI‐5 significantly improved neovascularization and blood flow recovery in experimental hind limb ischemia. Endothelial cells (ECs) derived from induced pluripotent stem cells (iPS‐ECs), iPS‐ECs OE‐QKI‐5 (iPS‐QKI‐5‐ECs) labeled with Vybrant, or phosphate buffer saline (PBS) as a control were injected intramuscularly immediately after induction of hind limb ischemia. **(A–C)**: Laser Doppler images of blood flow (BF) in the lower limbs of mice in prone position, with the ischemic leg highlighted by the white rectangle. **(D)**: Time course of BF recovery in the ischemic foot (calculated as a percentage ratio between ischemic foot BF and the contralateral foot BF) for each of the three conditions. Statistical analysis shows significantly higher BF recovery in the iPS‐EC QKI‐5 treated mice at 7 and 14 days in comparison with PBS control and also comparing iPS‐ECs to iPS‐ECs‐ OE‐QKI‐5; Bonferroni post test (to one way analysis of variance) confirms significant difference after 14 days between iPS‐ECs and iPS‐ECs overexpressing QKI‐5; *, *p* < .05; **, *p* < .01; ***, *p* < .001 (data are means ± SEM [*n* = 3]) **(E, F)**: Sections of adductor muscles were stained with CD144 or CD31 antibody and **(G)** capillary density expressed as capillary number per mm^2^, and **(E, F)** further co‐stained with CD144 (green) and Vybrant (red) prior to **(H)** quantification (and for CD31 please see Supporting Information Fig. S4A, S4B) *, *p* < .05; ***, *p* < .001 data are mean ± SEM, quantification from four random microscopic fields at ×40, scale bar = 50 μm). Abbreviations: CTL, control; EC, endothelial cell; iPSC, induced pluripotent stem cell; OE, overexpressing; PBS, phosphate buffered saline.

ECs derived from mouse iPSCs (iPS‐ECs CTL) or iPSC ECs OE‐QKI‐5 (iPS‐ECs OE‐QKI‐5) were labeled with Vybrant and injected intramuscularly immediately after induction of hind limb ischemia in mice. PBS was also used as an additional control. After 7 and 14 days, delivery of iPS‐ ECs OE‐QKI‐5 improved neovascularization and promoted significantly higher blood flow in the ischemic limbs compared to the non‐modified, iPS‐ECs controls or PBS controls (Fig. 5A–5D). Limbs receiving iPS‐ECs OE‐QKI‐5 also displayed significantly higher capillary numbers in the musculature in comparison with the controls (Fig. 5E, 5F) as shown by staining of adductor muscle sections with CD144 (Fig. 5G) and CD31 (Supporting Information Fig. S8A) in immunopositive vessel profiles. Notably, engrafted iPS‐ECs OE‐QKI‐5 displayed a typical vascular architecture, while in the PBS control experiments, a random pattern and no vascular structures were observed (Fig. 5E, 5F).

Finally, when adductor muscle sections from iPS‐ECs CTL or iPS‐ ECs OE‐QKI‐5 injected animals were stained and quantified with Vybrant and EC markers, iPS‐ ECs OE‐QKI‐5 were found to display an enhanced engraftment ability compared with controls (Fig. 5H; Supporting Information Fig. S8B). These results indicate that iPS‐ECs OE‐QKI‐5 display characteristic endothelial functions when tested in vivo. Importantly, no tumors have been detected in any animals during the duration of these experiments highlighting that these pluripotent stem cells were fully differentiated into ECs.

### QKI‐5 Has a Key Role in EC Differentiation Derived from hiPSCs

**Figure 6:**
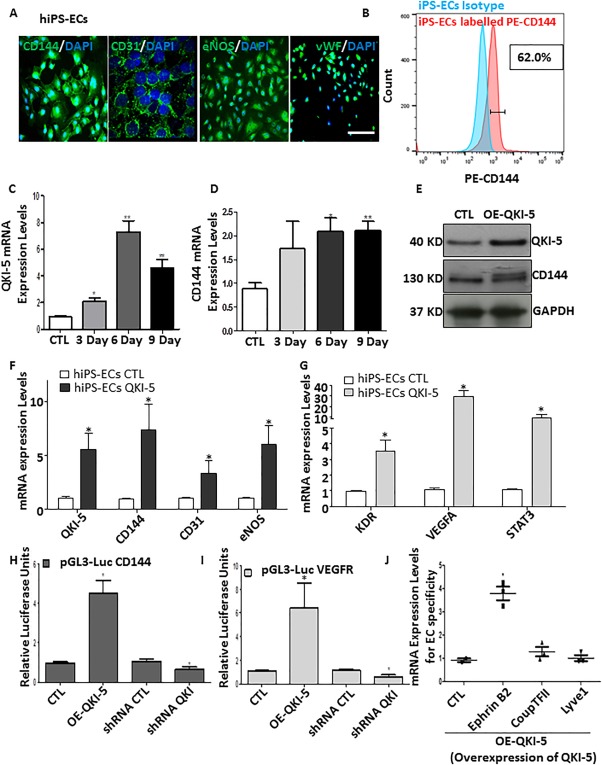
QKI‐5 has a key role in endothelial cell (EC) differentiation derived from human induced pluripotent stem cells (hiPSCs). **(A)**: hiPSCs were generated and differentiated on low attachment plates using StemPro serum free media supplemented with BMP4, Activin A, fibroblast growth factor, and vascular endothelial growth factor (VEGF) for 5 days. KDR (VEGFR) positive population was selected using MicroBeads and cultured on Fibronectin coated plates supplemented with EGM‐2 media for 3–9 days. iPS‐ECs‐derived cells expressed a typical pattern for EC specific markers CD144, CD31, eNOS, and vWF as immunofluorescence images shown. **(B)**: FACs analysis showing cells positive for EC specific marker CD144 **(C, D)** QKI‐5 was expressed during EC differentiation in parallel with VE‐cadherin expression in mRNA level. **(E–G)** QKI‐5 was overexpressed by lentiviral gene transfer in hiPSCs on day 3 after KDR selection and the cells were harvesting on day 6 of EC differentiation showing a significant induction of the EC markers CD144, CD31, and eNOS, and signaling of VEGFR (KDR), VEGFA, and STAT3 as Western blots and real time data shown. **(H, I)** Luciferase assays shown that QKI‐5 induced the transcriptional activation of CD144 and VEGFR. **(J)**: Overexpression of QKI‐5 directs differentiation toward arterial EC as cells express specific marker Ephrin B2 and not venous (CoupTFII) or lymphatic (Lyve1) EC markers (data are means ± SEM [*n* = 3]; *, *p* < .05; **, *p* < .01). Abbreviations: CTL, control; EC, endothelial cell; hiPSC, human induced pluripotent stem cell; iPSC, induced pluripotent stem cell.

Generation of high‐fidelity and stable EC populations, derived from human pluripotent cells, is a major limitation that the EC differentiation and cell reprogramming field are facing at the moment. Thus, we investigated whether QKI‐5 has a role in EC differentiation derived from hiPSCs. hiPSCs were generated and differentiated in low attachment plates using StemPro serum free media supplemented with BMP4, Activin A, FGF, and VEGF for 5 days. Then, a KDR positive population was selected and cultured in Fibronectin coated plates supplemented with EGM‐2 media for 3–9 days. The derived hiPSC‐ECs displayed a typical pattern of EC specific markers such as CD144, CD31, eNOS, and vWF (Fig. 6A). The efficiency of EC differentiation from hiPSCs was 62% as shown by FACs analysis for the positive cells for the EC marker CD144 (Fig. 6B). QKI‐5 was expressed during EC differentiation from hiPSCs in parallel with VE‐cadherin expression (Fig. 6C, 6D). QKI‐5 overexpression by lentiviral gene transfer significantly induced the expression of the EC markers CD144, CD31, and eNOS (Fig. 6E, 6F and signaling of VEGFR [KDR], VEGFA, and STAT3 during EC differentiation (Fig. 6G). Further data from luciferase assays also confirmed that QKI‐5 induced the transcriptional activation of CD144 and VEGFR in human cells (Fig. 6H, 6I). In contrast, knockdown of QKI‐5 by shRNA suppressed the transcriptional activation of both CD144 and VEGFR (Fig. 6H, 6I). Importantly, QKI‐5 only induced the expression of arterial specific EC marker and not the expression of venous and lymphatic markers (Fig. 6J). These data reveal that QKI‐5 is implicated in EC differentiation from hiPSCs toward an arterial EC specification.

### hiPSC‐ECs OE‐QKI‐5 Induced Angiogenesis in Vivo

**Figure 7:**
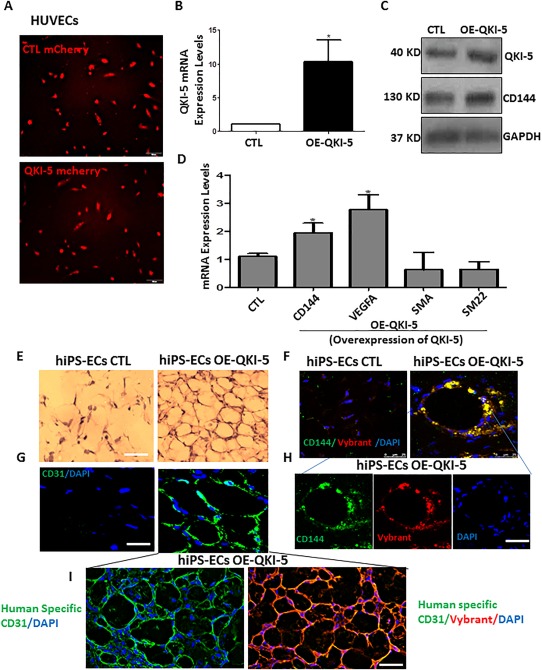
QKI‐5 induced CD144 and VEGFA expression in human umbilical vein endothelial cells (HUVECs) and Human iPS‐endothelial cells (ECs) overexpressing QKI‐5 induced angiogenesis in vivo. **(A)**: QKI‐5 was overexpressed in HUVECs by lentiviral gene transfer. The efficiency of infection was monitored by the mCherry expression. **(B, C)**: QKI‐5 was highly overexpressed in mature ECs and induced further the expression of CD144 and VEGFA **(C, D)**, but it did not induce non‐EC markers such as SMA and SM22 in the mRNA level **(D)** (data are means ± SEM. (*n* = 3); *, *p* < .05; **, *p* < .01). Scale bar = 50 µm. Human iPS‐ECs were forced to overexpress QKI‐5 by lentiviral gene transfer on day 3 of EC differentiation after KDR selection. On day 6, iPS‐ECs overexpressing an empty lentiviral vector or iPS‐QKI‐5‐ECs were labeled with Vybrant Cell Labeling and subcutaneously injected in SCID mice. **(E)**: iPS‐QKI‐5‐ECs significantly formed well‐defined vascular structures at 7 days in comparison with the control where less or none formed vascular structures were observed as H&E staining shown. **(F)**: Frozen section of CD31 immunofluorescence staining confirmed the presence of differentiated cells in in vivo vascular tubes. **(G, H)**: Enhanced engraftment ability of iPS‐QKI‐5‐ECs was observed when co‐stained with CD144 and Vybrant compared to the control iPS‐ECs. Scale bar = 50 µm. **(I)**: Further staining using human specific CD31 antibody confirms the presence of human cells and specificity of the Vybrant in samples seen in **(G, H)**. Abbreviations: CTL, control; DAPI, 4′,6‐Diamidine‐2′‐phenylindole dihydrochloride; EC, endothelial cell; hiPSC, human induced pluripotent stem cell; HUVEC, human umbilical vein endothelial cell.

As iPS‐ECs in the presence of QKI‐5 maintained a stable phenotype for many passages without drifting to various non‐endothelial phenotypes (as it has already been shown in Fig. 2), we further investigated whether QKI‐5 had a similar role in mature ECs. For these experiments, QKI‐5 was overexpressed in HUVECs by lentiviral gene transfer. These lentiviral constructs expressed mCherry, and the efficiency of infection was monitored by in vivo imaging using a fluorescence microscope (Fig. 7A). Indeed, QKI‐5 was highly overexpressed in these mature ECs (Fig. 7B) and was able to induce further the expression of CD144 and VEGFA in both protein and mRNA levels (Fig. 7C, 7D). Importantly, QKI‐5 overexpression did not induce the expression of non‐EC markers such as SMA and SM22 (Fig. 7C). These results highlight that QKI‐5 induces EC differentiation from hiPSCs and, in parallel, has an important role in the maintenance of the EC phenotype in both differentiated and mature ECs. Additional experiments with Matrigel plus assays in vivo provided further support to the above findings. hiPSC‐ECs OE‐QKI‐5 significantly induced angiogenesis only 7 days after subcutaneous injection of ECs OE‐ QKI‐5‐ iPSCs labeled with Vybrant cell surface marker in SCID mice, in comparison with the iPS‐ECs CTL expressing an empty lentiviral vector (HE staining in Fig. 7E). CD31 immunofluorescence staining confirmed the presence of differentiated cells in in vivo vascular tubes and demonstrated that QKI‐5 over‐expressing iPSCs displayed well‐formed vascular structures (Fig. 7G) and enhanced engraftment ability when co‐stained with CD144 and Vybrant compared with the control cells (Fig. 7F, 7H). In addition, a human specific antibody for CD31 was used to stain exclusively the human cells in the Matrigel plugs and co‐stained with the Vybrant to confirm the presence of the human cells and the specificity of the Vybrant labeling (Fig. 7I). These results clearly demonstrate that QKI‐5 induced angiogenesis in vivo Matrigel plug assays highlighting its pivotal role in EC differentiation derived from hiPSCs.

## Discussion

iPSCs represent an attractive cellular approach for regenerative medicine today as they can be used to generate therapeutic cells of almost any type and, importantly, can be harnessed as patient‐specific cells toward autologous cell therapy. In this study, using the model of iPSCs differentiation toward ECs, the RNA‐binding protein QKI‐5 was found to be an important regulator of VE‐cadherin stabilization and VEGFR2 transcriptional activation during the EC differentiation process. Notably, QKI‐5 overexpression induced the activation of EC markers, while knockdown by shRNA suppressed EC differentiation. In this study, the role of QKI‐5 has been elucidated in EC differentiation from iPSCs. iPSCs have been generated based on a highly efficient approach, fully characterized and forced to differentiate toward ECs. QKI‐5 was found to be induced during EC differentiation from iPSCs, and its expression was shown to be maintained at high levels in mature ECs. It has been demonstrated that QKI‐5 plays a role in the induction and stabilization of CD144 and activation of VEGFR‐regulatory binding sites AP1 and STAT3 and induction of STAT3 phosphorylation. Importantly, QKI‐5 modulated the activation of VEGFR through direct binding of the 3′ UTR region of STAT3. The notion that QKI‐5 indeed played an important role during EC differentiation was further supported from additional data which clearly demonstrated that knockdown of QKI‐5 resulted in inhibition of angiogenesis in vivo. Remarkably, ECs derived from iPS OE‐QKI‐5 improved neovascularization and blood flow recovery (almost 100%) in a hind limb ischemia model by showing an enhanced engraftment capacity when compared with non‐modified iPS‐ECs or PBS control groups. Notably, hiPSCs OE‐QKI‐5 induced angiogenesis in Matrigel plug assays in vivo only 7 days after subcutaneously injection in SCID mice, highlighting a clear functional benefit of QKI‐5 in neovascularization, blood flow recovery, and angiogenesis.

RNA‐binding proteins add an additional layer of complexity to a series of events, which include processing and splicing of pre‐mRNA, export to the cytoplasm, quality control assessment of mRNA through translation, message decay and stabilization, and translational repression and de‐repression 44. All of these events, from initiation of transcription by key transcription factors, to the stability and effective translation of the message, are regulated by the presence of specific nucleotide sequences which are bound by specific RNA‐binding proteins 45.

CD144 is a strictly endothelial specific adhesion molecule and is of vital importance in maintaining and controlling the endothelial phenotype through appropriate cell–cell contacts 16. CD144 is essential during embryonic angiogenesis and, thus, the elucidation of the underlying mechanisms that regulate CD144 are important in understanding the functions permeating vascular permeability, cell proliferation, apoptosis, and modulation of VEGFR. A recent study has shown that QKI directly binds to the 3′ UTR of CD144 46. In our study, we provide further support to the above notion that QKI‐5 induced the mRNA stabilization of CD144.

VEGFA is one of the earliest markers for the endothelial lineage during development 47, 48, and this growth factor is also critical for vascular repair and neovascularization 49. The data obtained in this study indicate that QKI‐5 regulates EC expression of VEGFA and autocrine stimulation of VEGFR and this pathway is controlled, at least in part, through STAT3 activation and phosphorylation. Interestingly, knockdown of STAT3 by shRNA ablated QKI‐5‐mediated activation of the VEGFR and subsequent expression of EC markers. It has been reported that constitutive STAT3 activity up‐regulates VEGFR 50, while STAT3 protein binds to the VEGFR promoter inducing VEGFR promoter activity 50. Interestingly, QKI‐5 regulates the expression of STAT3 through direct binding in the 3′UTR leading to mRNA stabilization. Notably, the KH DOMAIN‐mutant QKI‐5 construct did not induce the expression of the EC markers, STAT3 signaling when it was overexpressed in iPSCs. Importantly, QKI‐5 was unable to activate the 3′UTR of STAT3 when the QKI motif was deleted. These results demonstrate that QKI‐5 binds to the 3′UTR of STAT3 in the JAK1‐STAT3 signaling pathway and regulates EC differentiation toward an arterial lineage.

It has previously been reported that QKI‐5 knockout mice display a range of blood vessel defects during development which result in embryonic lethality 39. Additional studies of the extraembryonic yolk sac have also reported that QKI regulates visceral endoderm differentiated function at the cellular level, including the local synthesis of retinoic acid 29, which then exerts paracrine control of ECs within the adjacent mesoderm. QKI is also highly expressed during normal cardiac development, particularly in the outflow tract, suggesting potentially unique functions in the developing heart 51 and vascular smooth muscle cell development 38. Taken together, this is strong evidence that QKI is highly conserved in the early embryo throughout the evolution of nonvertebrate and vertebrate organisms 29. The role of QKI in the adult organism is less well‐appreciated, although there is evidence that it could have a role in vascular smooth muscle cell phenotypic plasticity and can ameliorate pathogenic, fibroproliferative responses to vascular injury 52.

This study provides strong evidence that QKI‐5 is capable of inducing differentiation toward ECs and that, in parallel, regulates angiogenesis in both in vitro and in vitro Matrigel plus assays, even in the absence of exogenous VEGF. At the moment, the potential of iPSCs to differentiate toward therapeutic cells is only based on directed empiricism, while they are totally dependent on combinations of growth factors, media, and matrices to favor the desired lineage. In regards to vascular regeneration, it is important to understand the key regulatory pathways such as epigenetic alterations, transcriptional activity, and RNA‐binding patterns associated with the differentiation processes. In particular, although there has been significant progression in the field 53, there are not any fully defined protocols to generate high‐fidelity and stable ECs from human pluripotent stem cells at the moment. This is a major limitation of generating pure populations of rejuvenated EC cells to be used for drug screening and cell‐based therapies. Indeed, QKI‐5 is a very interesting candidate, which holds the potential to derive stable populations of functional ECs. Specifically, the transcription factor ETS1 is implicated in the induction of the expression of QKI‐5 during early stages of EC differentiation. QKI‐5 is also acting as a splicing factor involved in the regulation of numerous signaling pathways. Data from our laboratory also showed that QKI‐5 induced the splicing factor SF3B1 during EC differentiation (Supporting Information Fig. S9). Importantly, SF3B1 is induced in a time‐dependent manner during EC differentiation from iPSCs (Supporting Information Fig. S10), indicating that QKI‐5 is likely to be an important splicing regulator of EC differentiation. Notably, a number of QKI splicing isoforms have been reported, although their function(s) are not clearly defined. Additional studies are clearly required to shed light on the precise functions of the different QKI splicing isoforms. Interestingly, a recent paper has reported that QKI plays a remarkably dynamic role in regulating hundreds of circular RNAs (circRNAs) during human epithelial to mesenchymal transition (EMT), while QKI itself is regulated during EMT 16. Therefore, it is tempting to speculate that precise regulation of QKI‐5 during EC differentiation will provide fully defined experimental protocols, which could reproducibly guide iPSCs to a vascular lineage 54, 55 and, thereby, enable clinical application 56, 57, 58. In addition, it would be very interesting to investigate the role of QKI and its mRNA splicing isoforms in disease models, such as atherosclerosis and diabetes, where the endothelial function is impaired.

## Summary

In summary, our data strongly support the notion that QKI‐5 is induced during EC differentiation, resulting in stabilization of STAT3 expression through direct binding to the 3′ UTR region of STAT3, modulation of VEGFR transcriptional activation and VEGF secretion. Markedly, iPS‐ECs OE‐QKI‐5 significantly improved angiogenesis and neovascularization and blood flow recovery in experimental hind limb ischemia (Supporting Information Fig. S9) highlighting a clear functional benefit. This study provides support to the growing consensus that elucidating the molecular mechanisms underlying EC differentiation will ultimately advance stem cell regenerative therapy toward treating cardiovascular disease.

## Author Contributions

A.C. and S.K.: conception and design, collection and/or assembly of data, data analysis and interpretation, manuscript writing; M.T., J.B., M.V.‐G., D.D., R.C., C.M., M.E., and Y.H.: collection and/or assembly of data; D.G., A.W.S., L.Z., and Q.X.: provision of study material, final approval of manuscript; A.M.: conception and design, collection and/or assembly of data, data analysis and interpretation, manuscript writing, financial support, final approval of manuscript. A.C. and S.K. contributed equally to this article.

## Disclosure of Potential Conflicts of Interest

The authors indicate no potential conflicts of interest.

## Supporting information


**Supplementary Figure S1: QKI is induced in parallel with CD144 in a pure population of differentiated ECs derived from mouse iPS cells.** (A‐B) Images taken of iPS‐ECs on days 7 and 10 of differentiation after VE‐cadherin positive selection demonstrate EC morphology, Scale bar, 50 μm. (C) Real time RT‐PCR data on day 10 during the expansion of this pure population of VE‐cadherin positive cells revealed that QKI is highly expressed in parallel with the endothelial marker VE‐cadherin, VEGFA and the transcription factors STAT3, [data are means ± SEM (n=3)] : * p<0.05, ** p<0.01, *** p<0.001.

**Supplementary Figure S2: QKI is induced in parallel with VE‐cadherin expression during EC differentiation from mouse Embryonic Stem cells (ESC Cells).** Further experiments were performed on mouse ESCs differentiated to ECs using a similar approach as the iPS cells. (A) Images of undifferentiated ESCs and (B) 8 days differentiated ESC‐ECs are shown, Scale bar, 50 μm. QKI expression was induced in parallel with CD144 expression during ESC to EC differentiation as real‐time RT‐PCR data revealed (C,D), (mean ±SEM, n=3, *, p<0.05).

**Supplementary Figure S3: QKI is capable of inducing EC differentiation from iPS cells by stabilising CD144** (A) iPS‐ECs were infected with QKI or an empty vector on day 4 of EC differentiation. Two days later the cells were treated with actinomycin D (1μg/ml) for 6 h or cyclohexamide for 24 hours, western blots show that VE‐Cadherin expression was stabilised In the presence of QKI (A,C with quantification B,D). (E) When differentiated ECs were treated with Actinomycin D in a time point experiment from 0 to 24 hours CD144 expression was stabilised as a decay curve is shown (mean ±SEM, n=3, *, p<0.05), (mean ±SEM, n=3, *, p<0.05).

**Supplementary Figure S4: QKI is capable of initiating EC differentiation from iPS cells** QKI was overexpressed in undifferentiated iPS and 3 days later the cells were harvested, RNA was extracted and analyzed by real time PCR. (A) The data reveal that the cells expressed arterial marker Ephrin B2 but no venous (CoupTF11), lymphatic (Lyve1) EC markers or markers of other lineages such as Nestin and SMA. (B) EC makers were also highly induced after 3 days of QKI overexpression in undifferentiated iPS). (C) ETS1 expression is induced during EC differentiation from iPS cells in a time‐dependent manner, and (D) overexpression of ETS1 (pGEM‐ETS1: Sino Biological Inc. #HG12103‐G) via transfection in early EC differentiation from iPS cells leads to the induction of QKI (H), (data are means ± SEM. (n=3), *p<0.05).

**Supplementary Figure S5: QKI‐5 induced VEGF Receptor 2 (VEGFR2) activation and EC differentiation through STAT3 /JAK1 signaling** (A) Luciferase assays show that when STAT3 was knocked down by shRNA on day 3 and next day QKI‐5 was overexpressed the transcriptional activation of the VEGFR2‐mediated by QKI‐5 was ablated. For these experiments QKI‐5 was overexpressed on day 4 and the cells were harvested on day 6 prior to Luciferase analysis. (B) When the differentiated ECs were treated with inhibitors on day 3 of differentiation, to block the JAK‐1 (S2219, 10 μM), and STAT3 (S1155, 250 μM) pathways, QKI‐5 did not activate the EC markers CD31, CD144, eNOS, and FLK‐1 (VEGFR2) and VEGFA at day 6, when overexpressed on day 4. (data are means ± SEM. (n=3), *p<0.05).

**Supplementary Figure S6: The RNA binding domain (KH Domain) is required for QKI function in EC differentiation** (A) Schematic showing QKI structure and localisation of KH domain and experimental design for mutant construct where primers were designed to loop out the domain. When a KH DOMAIN‐mutant QKI construct was overexpressed in the iPS cells (B) QKI no longer induced the expression of the EC markers, and STAT3 signalling (C) (mean ±SEM, n=3, *, p<0.05).

**Supplementary Figure S7: Nascent vessels in vivo (matrigel plugs) were stabilised by pericytes/vascular smooth muscle cells in the presence of QKI‐5.** Differentiated ECs overexpressing QKI‐5 were subjected to Matrigel plug assays *in vivo*. Frozen sections from the *in vivo* Matrigel plugs were stained for Smooth Muscle Actin (SMA) to demonstrate that the nascent vessels in vivo (matrigel plugs) were stabilised by pericytes/vascular smooth muscle cells in the presence of QKI‐5 as smooth muscle alpha‐ actin (SMA) staining is shown, Scale bar, 50 μm.

**Supplementary Figure S8: QKI significantly improved neovascularization and blood flow recovery in experimental hind limb ischemia.** ECs derived from iPS cells (iPS‐ECs), iPS‐ECs overexpressing QKI (iPS‐ECs‐QKI) labeled with Vybrant, or PBS as a control were injected intramuscularly immediately after induction of hind limb ischemia. (A) Sections of adductor muscles were stained with CD31 antibody and capillary density expressed as capillary number per mm2, with (B) further co‐staining with CD31(green) and Vybrant (red) (data are means ± SEM * p<0.05, ** p<0.01, quantification from 4 random microscopic fields at x40, scale: 50μm).

**Supplementary Figure S9: QKI regulates the splicing factor SF3B1 during EC differentiation.** Overexpression experiments show that QKI is implicated in the regulation of the splicing factor SF3B1, on day 6 of EC differentiation from iPS cells, as assessed by real‐time RT‐RCR (data are means ± SEM n=3 * p<0.05).

**Supplementary Figure S10: The splicing factor SF3B1 is induced during iPS cells differentiation.** (A) The spicing factor SF3B1 is induced in a time dependent manner during EC differentiation derived from iPS cells as it is shown by real time data,, (* p<0.05, ** p<0.01, *** p<0.001 (data are means ± SEM (n=3)).

**Supplementary Figure S11: Schematic illustration of the identified role of QKI as a key regulator of EC differentiation and angiogenesis.** QKI is induced during EC differentiation downstream of ETS1, resulting in stabilisation of VE‐cadherin expression and modulation of VEGFR transcriptional activation and VEGF secretion, through direct binding to the 3' UTR of STAT3, which leads to its activation. iPS‐ECs overexpressing QKI significantly improved angiogenesis, neovascularization and blood flow recovery in experimental hind limb ischemia.

Supporting Information 1

Supporting Information 2

## References

[1] Cines DB , Pollak ES , Buck CA et al. Endothelial cells in physiology and in the pathophysiology of vascular disorders. Blood 1998;91:3527–3561. [Database] 9572988

[2] Davignon J Ganz P. Role of endothelial dysfunction in atherosclerosis. Circulation 2004;109:III27–III32. 15198963 10.1161/01.CIR.0000131515.03336.f8

[3] Collado M , Blasco MA Serrano M. Cellular senescence in cancer and aging. Cell 2007;130:223–233. 17662938 10.1016/j.cell.2007.07.003

[4] Cheng Y , Ji R , Yue J et al. MicroRNAs are aberrantly expressed in hypertrophic heart: Do they play a role in cardiac hypertrophy? Am J Pathol 2007;170:1831–1840. 17525252 10.2353/ajpath.2007.061170 PMC1899438

[5] Sayed D , Hong C , Chen IY et al. MicroRNAs play an essential role in the development of cardiac hypertrophy. Circ Res 2007;100:416–424. 17234972 10.1161/01.RES.0000257913.42552.23

[6] Yi F , Liu GH Belmonte JC. Rejuvenating liver and pancreas through cell transdifferentiation. Cell Res 2012;22:616–619. 22373548 10.1038/cr.2012.33 PMC3317567

[7] Wong WT , Huang NF , Botham CM et al. Endothelial cells derived from nuclear reprogramming. Circ Res 2012;111:1363–1375. 23104878 10.1161/CIRCRESAHA.111.247213 PMC3526979

[8] Reed DM , Foldes G , Harding SE et al. Stem cell derived endothelial cells for cardiovascular disease: A therapeutic perspective. Br J Clin Pharmacol 2013;75:897–906. 22703602 10.1111/j.1365-2125.2012.04361.x PMC3612707

[9] Ferreira LS , Gerecht S , Shieh HF et al. Vascular progenitor cells isolated from human embryonic stem cells give rise to endothelial and smooth muscle like cells and form vascular networks in vivo. Circ Res 2007;101:286–294. 17569886 10.1161/CIRCRESAHA.107.150201

[10] Takahashi K , Yamanaka S. Induction of pluripotent stem cells from mouse embryonic and adult fibroblast cultures by defined factors. Cell 2006;126:663–676. 16904174 10.1016/j.cell.2006.07.024

[11] Bonauer A , Carmona G , Iwasaki M et al. MicroRNA‐92a controls angiogenesis and functional recovery of ischemic tissues in mice. Science 2009;324:1710–1713. 19460962 10.1126/science.1174381

[12] Small EM , Frost RJ , Olson EN. MicroRNAs add a new dimension to cardiovascular disease. Circulation 2010;121:1022–1032. 20194875 10.1161/CIRCULATIONAHA.109.889048 PMC2847432

[13] Rufaihah AJ , Huang NF , Kim J et al. Human induced pluripotent stem cell‐derived endothelial cells exhibit functional heterogeneity. Am J Trans Res 2013;5:21–35. PMC3560482 23390563

[14] Azhdari M , Baghaban‐Eslaminejad M , Baharvand H et al. Therapeutic potential of human‐induced pluripotent stem cell‐derived endothelial cells in a bleomycin‐induced scleroderma mouse model. Stem Cell Res 2013;10:288–300. 23396195 10.1016/j.scr.2012.12.004

[15] Tolar J , Park IH , Xia L et al. Hematopoietic differentiation of induced pluripotent stem cells from patients with mucopolysaccharidosis type I (Hurler syndrome). Blood 2011;117:839–847. 21037085 10.1182/blood-2010-05-287607 PMC3035077

[16] Conn SJ , Pillman KA , Toubia J et al. The RNA binding protein quaking regulates formation of circRNAs. Cell 2015;160:1125–1134. 25768908 10.1016/j.cell.2015.02.014

[17] Ring KL , Tong LM , Balestra ME et al. Direct reprogramming of mouse and human fibroblasts into multipotent neural stem cells with a single factor. Cell Stem Cell 2012;11:100–109. 22683203 10.1016/j.stem.2012.05.018 PMC3399516

[18] Li R , Liang J , Ni S et al. A mesenchymal‐to‐epithelial transition initiates and is required for the nuclear reprogramming of mouse fibroblasts. Cell Stem Cell 2010;7:51–63. 20621050 10.1016/j.stem.2010.04.014

[19] Park IH , Zhao R , West JA et al. Reprogramming of human somatic cells to pluripotency with defined factors. Nature 2008;451:141–146. 18157115 10.1038/nature06534

[20] Kelaini S , Cochrane A , Margariti A. Direct reprogramming of adult cells: Avoiding the pluripotent state. Stem Cell Clon Adv Appl 2014;7:19–29. 10.2147/SCCAA.S38006 PMC3931695 24627642

[21] Margariti A , Winkler B , Karamariti E et al. Direct reprogramming of fibroblasts into endothelial cells capable of angiogenesis and reendothelialization in tissue‐engineered vessels. Proc Natl Acad Sci U S A 2012;109:13793–13798. 22869753 10.1073/pnas.1205526109 PMC3427074

[22] Li J , Huang NF , Zou J et al. Conversion of human fibroblasts to functional endothelial cells by defined factors. Arterioscler Thromb Vasc Biol 2013;33:1366–1375. 23520160 10.1161/ATVBAHA.112.301167 PMC3898631

[23] Wang G , Guo X , Hong W et al. Critical regulation of miR‐200/zeb2 pathway in oct4/sox2‐induced mesenchymal‐to‐epithelial transition and induced pluripotent stem cell generation. Proc Natl Acad Sci U S A 2013;110:2858–2863. 23386720 10.1073/pnas.1212769110 PMC3581874

[24] Torres A , Torres K , Pesci A et al. Deregulation of miR‐100, miR‐99a and miR‐199b in tissues and plasma coexists with increased expression of mTOR kinase in endometrioid endometrial carcinoma. BMC Cancer 2012;12:369. 22920721 10.1186/1471-2407-12-369 PMC3495850

[25] Yoo AS , Sun AX , Li L et al. MicroRNA‐mediated conversion of human fibroblasts to neurons. Nature 2011;476:228–231. 21753754 10.1038/nature10323 PMC3348862

[26] Sekiya S , Suzuki A. Direct conversion of mouse fibroblasts to hepatocyte‐like cells by defined factors. Nature 2011;475:390–393. 21716291 10.1038/nature10263

[27] Huang NF , Li S. Regulation of the matrix microenvironment for stem cell engineering and regenerative medicine. Ann Biomed Eng 2011;39:1201–1214. 21424849 10.1007/s10439-011-0297-2 PMC3568678

[28] Margariti A , Xiao Q , Zampetaki A et al. Splicing of HDAC7 modulates the SRF‐myocardin complex during stem‐cell differentiation towards smooth muscle cells. J Cell Sci 2009;122:460–470. 19174469 10.1242/jcs.034850

[29] Justice MJ Hirschi KK. The role of quaking in mammalian embryonic development. Adv Exp Med Biol 2010;693:82–92. 21189687 10.1007/978-1-4419-7005-3_6

[30] Shang W , Chen X , Nie L et al. Mir199b suppresses expression of hypoxia‐inducible factor 1alpha (hif‐1alpha) in prostate cancer cells. Int J Mol Sci 2013;14:8422–8436. 23594994 10.3390/ijms14048422 PMC3645752

[31] Carmeliet P. Angiogenesis in health and disease. Nat Med 2003;9:653–660. 12778163 10.1038/nm0603-653

[32] Greco S , Fasanaro P , Castelvecchio S et al. MicroRNA dysregulation in diabetic ischemic heart failure patients. Diabetes 2012;61:1633–1641. 22427379 10.2337/db11-0952 PMC3357263

[33] Pober JS Sessa WC. Evolving functions of endothelial cells in inflammation. Nat Rev Immunol 2007;7:803–815. 17893694 10.1038/nri2171

[34] da Costa Martins PA , Salic K , Gladka MM et al. MicroRNA‐199b targets the nuclear kinase Dyrk1a in an auto‐amplification loop promoting calcineurin/nfat signalling. Nature Cell Biology 2010;12:1220–1227. 21102440 10.1038/ncb2126

[35] Kuehbacher A , Urbich C , Zeiher AM et al. Role of dicer and drosha for endothelial microRNA expression and angiogenesis. Circ Res 2007;101:59–68. 17540974 10.1161/CIRCRESAHA.107.153916

[36] Chenard CA Richard S. New implications for the quaking RNA binding protein in human disease. J Neurosci Res 2008;86:233–242. 17787018 10.1002/jnr.21485

[37] Hardy RJ , Loushin CL , Friedrich VL Jr et al. Neural cell type‐specific expression of qkI proteins is altered in quaking viable mutant mice. J Neurosci 1996;16:7941–7949. 8987822 10.1523/JNEUROSCI.16-24-07941.1996 PMC6579212

[38] Li Z , Takakura N , Oike Y et al. Defective smooth muscle development in qkI‐deficient mice. Dev Growth Differ 2003;45:449–462. 14706070 10.1111/j.1440-169x.2003.00712.x

[39] Noveroske JK , Lai L , Gaussin V et al. Quaking is essential for blood vessel development. Genesis 2002;32:218–230. 11892011 10.1002/gene.10060

[40] Di Bernardini E , Campagnolo P , Margariti A et al. Endothelial lineage differentiation from induced pluripotent stem cells is regulated by microRNA‐21 and transforming growth factor beta2 (tgf‐beta2) pathways. J Biol Chem 2014;289:3383–3393. 24356956 10.1074/jbc.M113.495531 PMC3916541

[41] Galarneau A , Richard S. Target RNA motif and target mRNAs of the quaking star protein. Nature Structural & Molecular Biol 2005;12:691–698. 10.1038/nsmb963 16041388

[42] Emanueli C , Monopoli A , Kraenkel N et al. Nitropravastatin stimulates reparative neovascularisation and improves recovery from limb ischaemia in type‐1 diabetic mice. Br J Pharmacol 2007;150:873–882. 17351667 10.1038/sj.bjp.0707142 PMC2013882

[43] Zhou Y , Yang F , Chen T et al. An updated view on the differentiation of stem cells into endothelial cells. Science China. Life Sci 2014;57:763–773. 10.1007/s11427-014-4712-4 25104448

[44] Whelan JT , Hollis SE , Cha DS et al. Post‐transcriptional regulation of the ras‐erk/mapk signaling pathway. J Cell Physiol 2012;227:1235–1241. 21688267 10.1002/jcp.22899

[45] Glisovic T , Bachorik JL , Yong J et al. RNA‐binding proteins and post‐transcriptional gene regulation. FEBS Lett 2008;582:1977–1986. 18342629 10.1016/j.febslet.2008.03.004 PMC2858862

[46] de Bruin RG , van der Veer EP , Prins J et al. The RNA‐binding protein quaking maintains endothelial barrier function and affects VE‐cadherin and beta‐catenin protein expression. Sci Rep 2016;6:21643. 26905650 10.1038/srep21643 PMC4764852

[47] Carmeliet P , Ferreira V , Breier G et al. Abnormal blood vessel development and lethality in embryos lacking a single VEGF allele. Nature 1996;380:435–439. 8602241 10.1038/380435a0

[48] Carmeliet P , Lampugnani MG , Moons L et al. Targeted deficiency or cytosolic truncation of the VE‐cadherin gene in mice impairs VEGF‐mediated endothelial survival and angiogenesis. Cell 1999;98:147–157. 10428027 10.1016/s0092-8674(00)81010-7

[49] Gianni‐Barrera R , Bartolomeo M , Vollmar B et al. Split for the cure: VEGF, PDGF‐BB and intussusception in therapeutic angiogenesis. Biochem Soc Trans 2014;42:1637–1642. 25399582 10.1042/BST20140234

[50] Niu G , Wright KL , Huang M et al. Constitutive stat3 activity up‐regulates VEGF expression and tumor angiogenesis. Oncogene 2002;21:2000–2008. 11960372 10.1038/sj.onc.1205260

[51] van Mil A , Grundmann S , Goumans MJ et al. MicroRNA‐214 inhibits angiogenesis by targeting quaking and reducing angiogenic growth factor release. Cardiovasc Res 2012;93:655–665. 22227154 10.1093/cvr/cvs003

[52] van der Veer EP , de Bruin RG , Kraaijeveld AO et al. Quaking, an RNA‐binding protein, is a critical regulator of vascular smooth muscle cell phenotype. Circ Res 2013;113:1065–1075. 23963726 10.1161/CIRCRESAHA.113.301302

[53] Lian X , Bao X , Al‐Ahmad A et al. Efficient differentiation of human pluripotent stem cells to endothelial progenitors via small‐molecule activation of WNT signaling. Stem Cell Reports 2014;3:804–816. 25418725 10.1016/j.stemcr.2014.09.005 PMC4235141

[54] Mauritz C , Schwanke K , Reppel M et al. Generation of functional murine cardiac myocytes from induced pluripotent stem cells. Circulation 2008;118:507–517. 18625890 10.1161/CIRCULATIONAHA.108.778795

[55] Nishikawa S , Goldstein RA , Nierras CR. The promise of human induced pluripotent stem cells for research and therapy. Nat Rev Mol Cell Biol 2008;9:725–729. 18698329 10.1038/nrm2466

[56] Asahara T , Kawamoto A. Endothelial progenitor cells for postnatal vasculogenesis. Am J Physiol Cell Physiol 2004;287:C572–C579. 15308462 10.1152/ajpcell.00330.2003

[57] Zhao R , Daley GQ. From fibroblasts to iPSCs: Induced pluripotency by defined factors. J Cell Biochem 2008;105:949–955. 18668528 10.1002/jcb.21871

[58] Zhang L , Zhou J , Lu Q et al. A novel small‐diameter vascular graft: In vivo behavior of biodegradable three‐layered tubular scaffolds. Biotechnol Bioeng 2008;99:1007–1015. 17705246 10.1002/bit.21629

