# Supplementary figures and images for: Quaking Is a Key Regulator of Endothelial Cell Differentiation, Neovascularization, and Angiogenesis
Source: Stem Cells. 2017 Mar 5;35(4):952–66. doi: 10.1002/stem.2594 (PMC5396345; doi:10.1002/stem.2594)

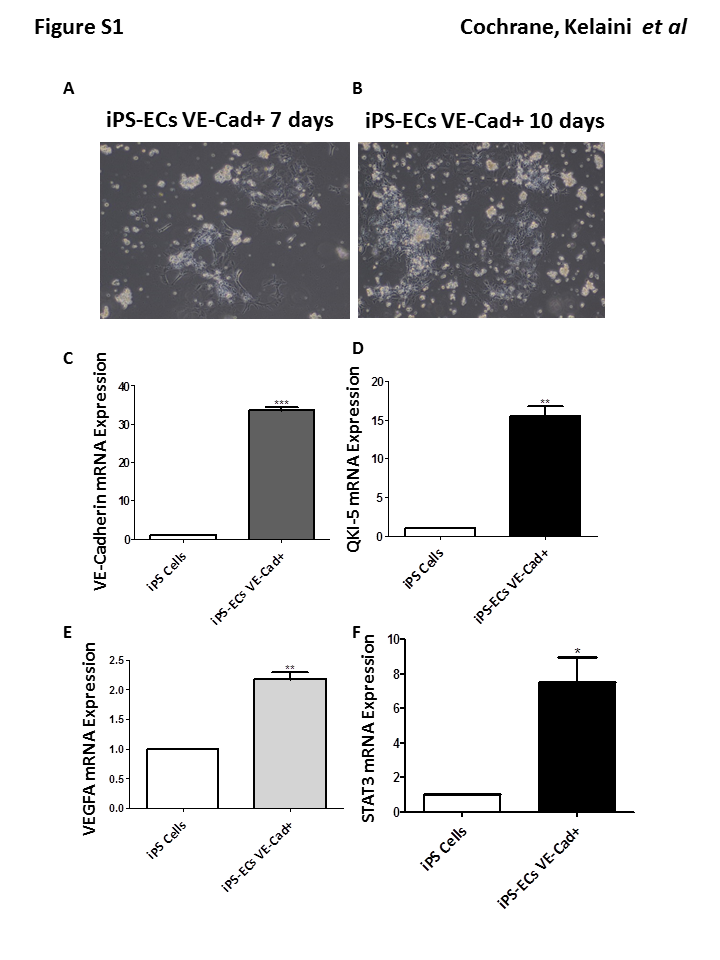

Supplement: Supplementary file 1 — Supplementary Figure S1: QKI is induced in parallel with CD144 in a pure population of differentiated ECs derived from mouse iPS cells. (A‐B) Images taken of iPS‐ECs on days 7 and 10 of differentiation after VE‐cadherin positive selection demonstrate EC morphology, Scale bar, 50 μm. (C) Real time RT‐PCR data on day 10 during the expansion of this pure population of VE‐cadherin positive cells revealed that QKI is highly expressed in parallel with the endothelial marker VE‐cadherin, VEGFA and the transcription factors STAT3, [data are means ± SEM (n=3)] : * p<0.05, ** p<0.01, *** p<0.001. [file STEM-35-952-s001.TIF]

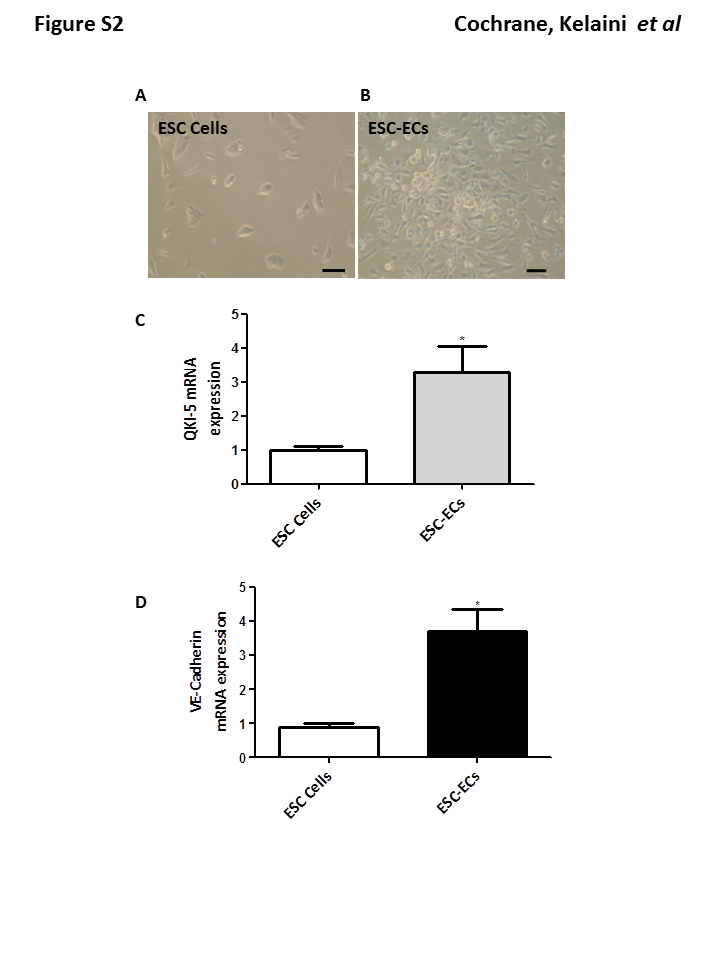

Supplement: Supplementary file 2 — Supplementary Figure S2: QKI is induced in parallel with VE‐cadherin expression during EC differentiation from mouse Embryonic Stem cells (ESC Cells). Further experiments were performed on mouse ESCs differentiated to ECs using a similar approach as the iPS cells. (A) Images of undifferentiated ESCs and (B) 8 days differentiated ESC‐ECs are shown, Scale bar, 50 μm. QKI expression was induced in parallel with CD144 expression during ESC to EC differentiation as real‐time RT‐PCR data revealed (C,D), (mean ±SEM, n=3, *, p<0.05). [file STEM-35-952-s002.TIF]

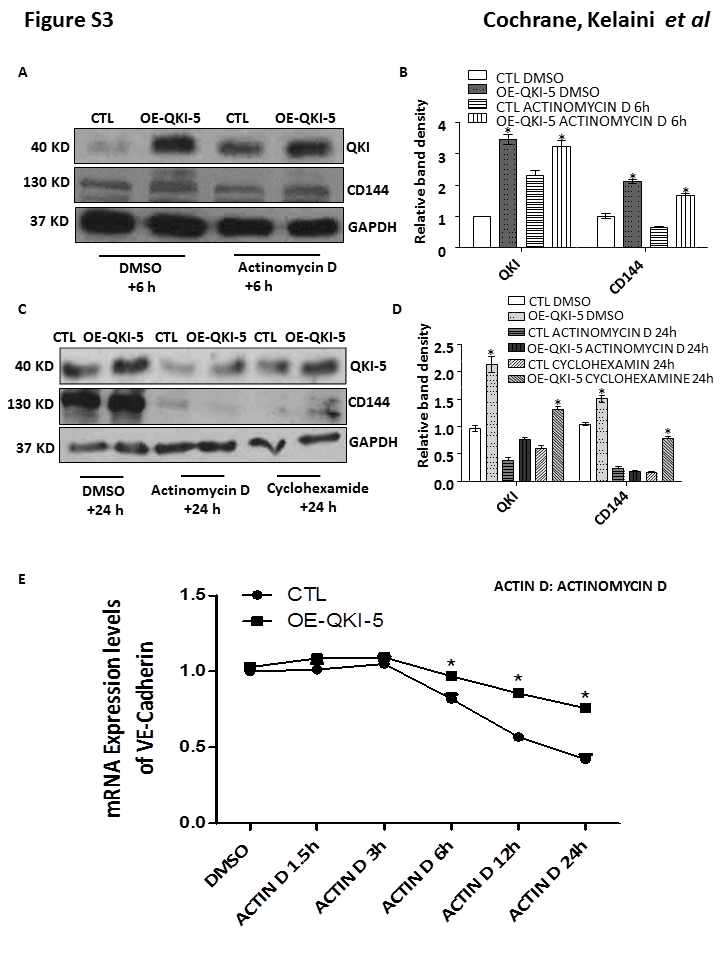

Supplement: Supplementary file 3 — Supplementary Figure S3: QKI is capable of inducing EC differentiation from iPS cells by stabilising CD144 (A) iPS‐ECs were infected with QKI or an empty vector on day 4 of EC differentiation. Two days later the cells were treated with actinomycin D (1μg/ml) for 6 h or cyclohexamide for 24 hours, western blots show that VE‐Cadherin expression was stabilised In the presence of QKI (A,C with quantification B,D). (E) When differentiated ECs were treated with Actinomycin D in a time point experiment from 0 to 24 hours CD144 expression was stabilised as a decay curve is shown (mean ±SEM, n=3, *, p<0.05), (mean ±SEM, n=3, *, p<0.05). [file STEM-35-952-s003.TIF]

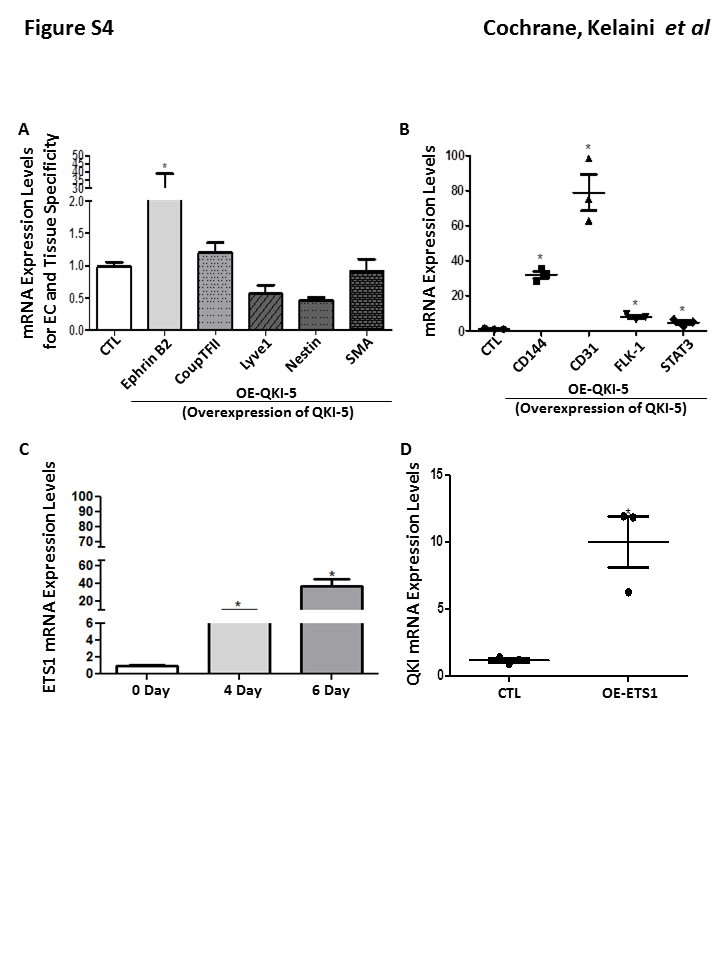

Supplement: Supplementary file 4 — Supplementary Figure S4: QKI is capable of initiating EC differentiation from iPS cells QKI was overexpressed in undifferentiated iPS and 3 days later the cells were harvested, RNA was extracted and analyzed by real time PCR. (A) The data reveal that the cells expressed arterial marker Ephrin B2 but no venous (CoupTF11), lymphatic (Lyve1) EC markers or markers of other lineages such as Nestin and SMA. (B) EC makers were also highly induced after 3 days of QKI overexpression in undifferentiated iPS). (C) ETS1 expression is induced during EC differentiation from iPS cells in a time‐dependent manner, and (D) overexpression of ETS1 (pGEM‐ETS1: Sino Biological Inc. #HG12103‐G) via transfection in early EC differentiation from iPS cells leads to the induction of QKI (H), (data are means ± SEM. (n=3), *p<0.05). [file STEM-35-952-s004.TIF]

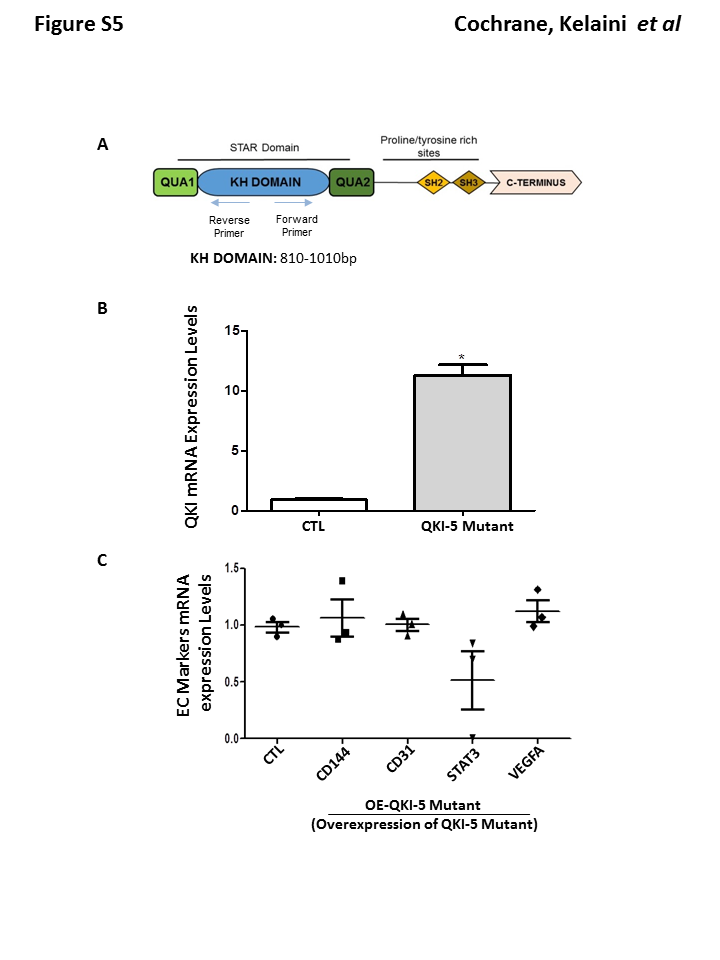

Supplement: Supplementary file 5 — Supplementary Figure S5: QKI‐5 induced VEGF Receptor 2 (VEGFR2) activation and EC differentiation through STAT3 /JAK1 signaling (A) Luciferase assays show that when STAT3 was knocked down by shRNA on day 3 and next day QKI‐5 was overexpressed the transcriptional activation of the VEGFR2‐mediated by QKI‐5 was ablated. For these experiments QKI‐5 was overexpressed on day 4 and the cells were harvested on day 6 prior to Luciferase analysis. (B) When the differentiated ECs were treated with inhibitors on day 3 of differentiation, to block the JAK‐1 (S2219, 10 μM), and STAT3 (S1155, 250 μM) pathways, QKI‐5 did not activate the EC markers CD31, CD144, eNOS, and FLK‐1 (VEGFR2) and VEGFA at day 6, when overexpressed on day 4. (data are means ± SEM. (n=3), *p<0.05). [file STEM-35-952-s005.TIF]

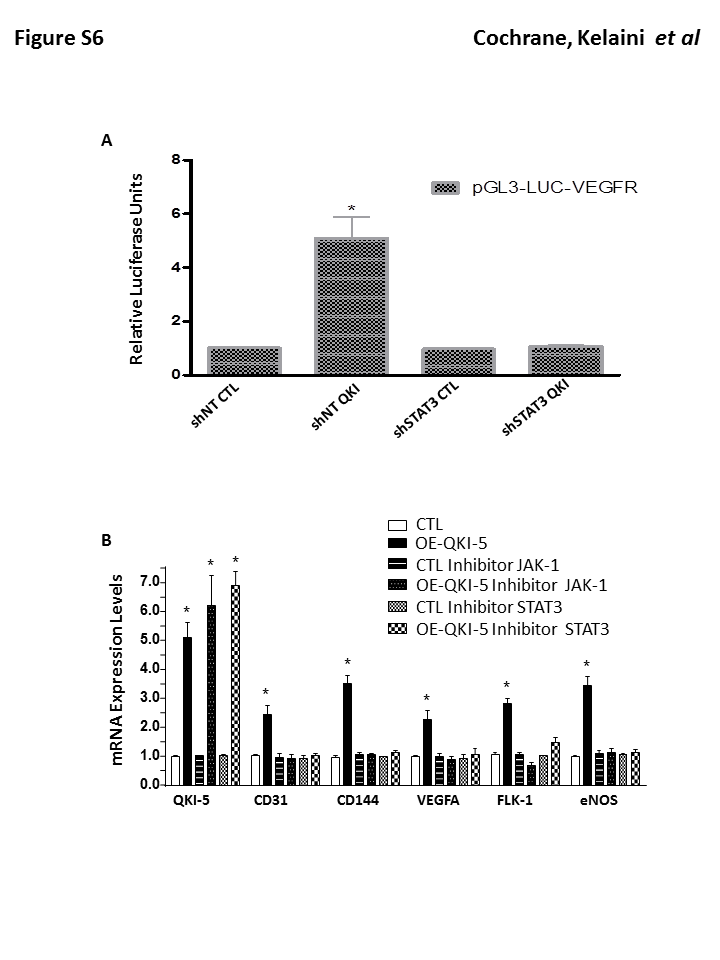

Supplement: Supplementary file 6 — Supplementary Figure S6: The RNA binding domain (KH Domain) is required for QKI function in EC differentiation (A) Schematic showing QKI structure and localisation of KH domain and experimental design for mutant construct where primers were designed to loop out the domain. When a KH DOMAIN‐mutant QKI construct was overexpressed in the iPS cells (B) QKI no longer induced the expression of the EC markers, and STAT3 signalling (C) (mean ±SEM, n=3, *, p<0.05). [file STEM-35-952-s006.TIF]

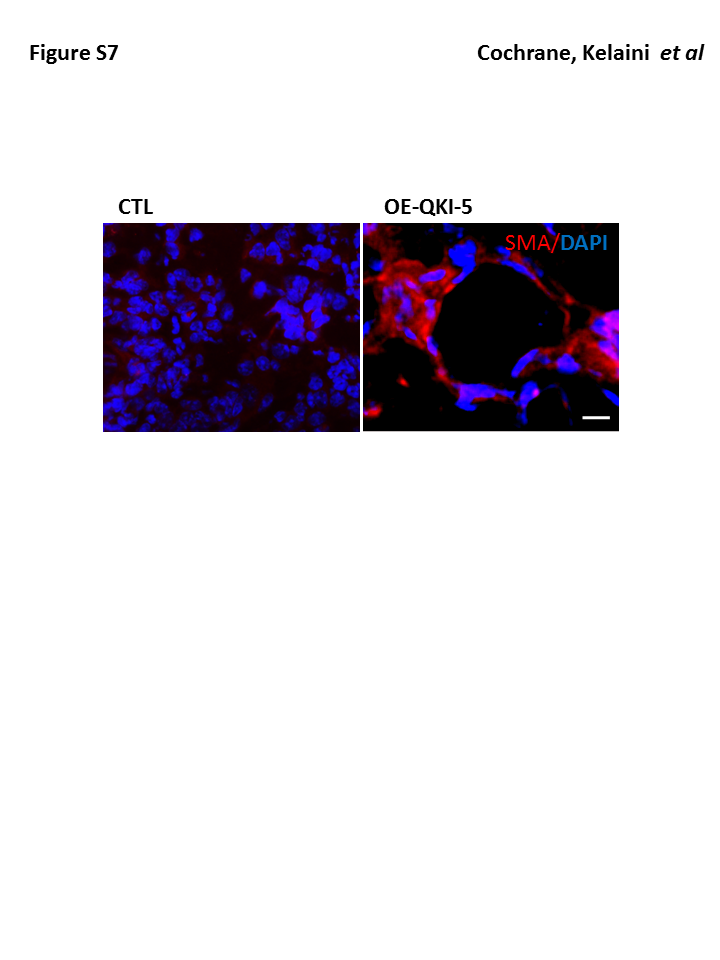

Supplement: Supplementary file 7 — Supplementary Figure S7: Nascent vessels in vivo (matrigel plugs) were stabilised by pericytes/vascular smooth muscle cells in the presence of QKI‐5. Differentiated ECs overexpressing QKI‐5 were subjected to Matrigel plug assays in vivo. Frozen sections from the in vivo Matrigel plugs were stained for Smooth Muscle Actin (SMA) to demonstrate that the nascent vessels in vivo (matrigel plugs) were stabilised by pericytes/vascular smooth muscle cells in the presence of QKI‐5 as smooth muscle alpha‐ actin (SMA) staining is shown, Scale bar, 50 μm. [file STEM-35-952-s007.TIF]

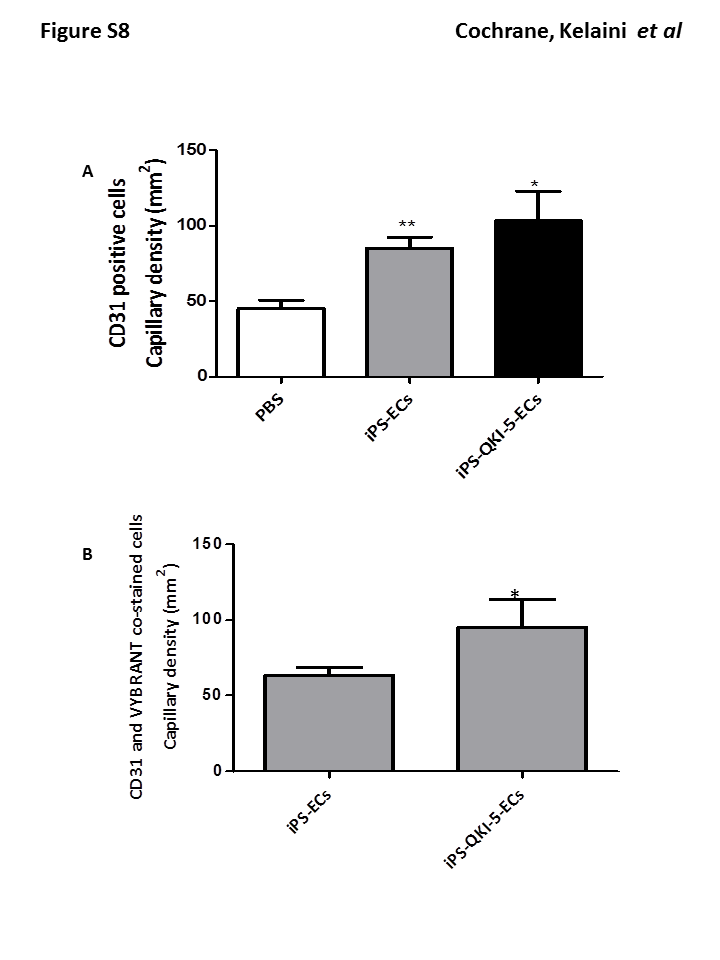

Supplement: Supplementary file 8 — Supplementary Figure S8: QKI significantly improved neovascularization and blood flow recovery in experimental hind limb ischemia. ECs derived from iPS cells (iPS‐ECs), iPS‐ECs overexpressing QKI (iPS‐ECs‐QKI) labeled with Vybrant, or PBS as a control were injected intramuscularly immediately after induction of hind limb ischemia. (A) Sections of adductor muscles were stained with CD31 antibody and capillary density expressed as capillary number per mm2, with (B) further co‐staining with CD31(green) and Vybrant (red) (data are means ± SEM * p<0.05, ** p<0.01, quantification from 4 random microscopic fields at x40, scale: 50μm). [file STEM-35-952-s008.TIF]

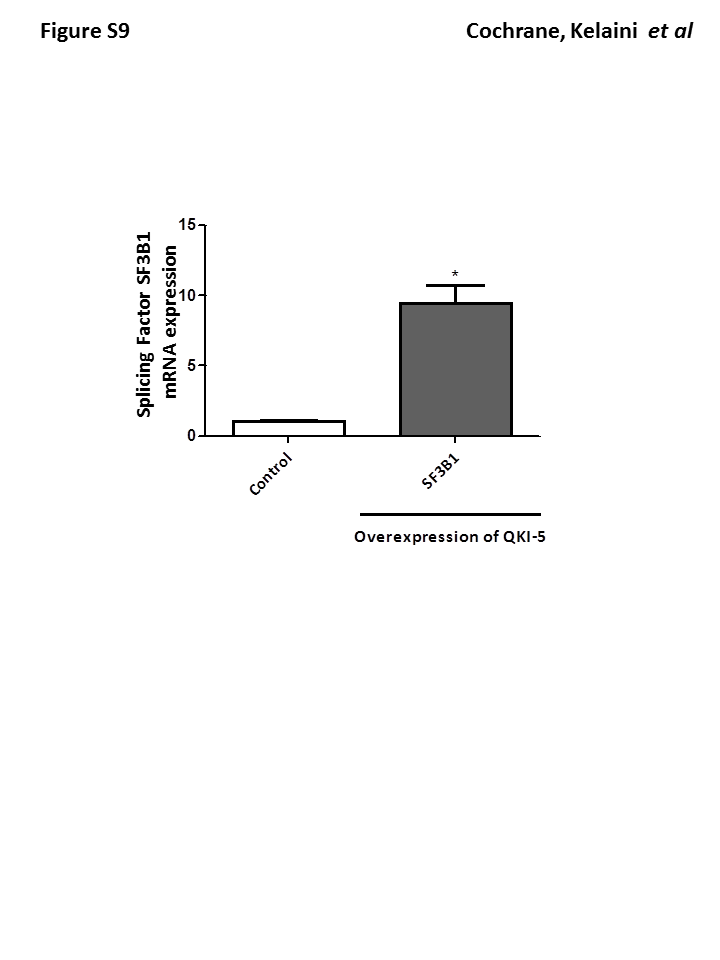

Supplement: Supplementary file 9 — Supplementary Figure S9: QKI regulates the splicing factor SF3B1 during EC differentiation. Overexpression experiments show that QKI is implicated in the regulation of the splicing factor SF3B1, on day 6 of EC differentiation from iPS cells, as assessed by real‐time RT‐RCR (data are means ± SEM n=3 * p<0.05). [file STEM-35-952-s009.TIF]

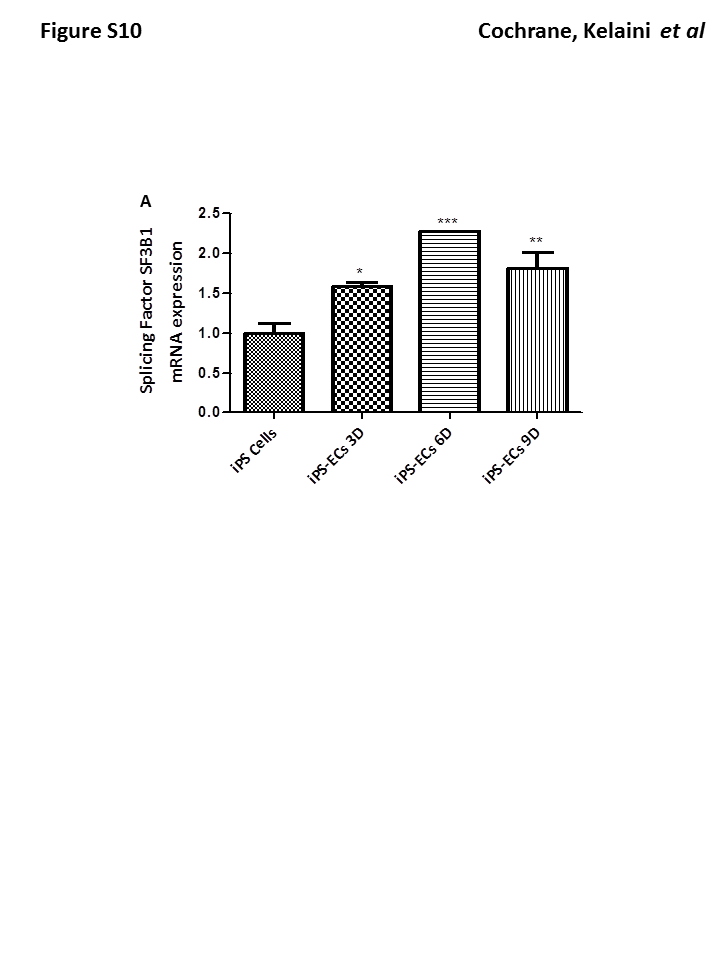

Supplement: Supplementary file 10 — Supplementary Figure S10: The splicing factor SF3B1 is induced during iPS cells differentiation. (A) The spicing factor SF3B1 is induced in a time dependent manner during EC differentiation derived from iPS cells as it is shown by real time data,, (* p<0.05, ** p<0.01, *** p<0.001 (data are means ± SEM (n=3)). [file STEM-35-952-s010.TIF]

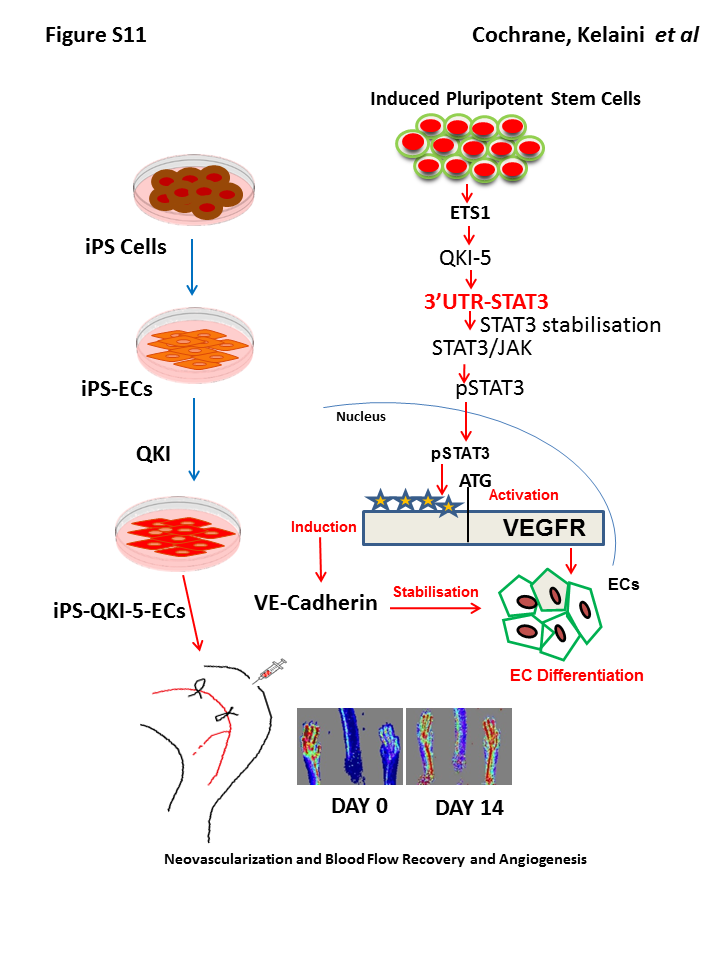

Supplement: Supplementary file 11 — Supplementary Figure S11: Schematic illustration of the identified role of QKI as a key regulator of EC differentiation and angiogenesis. QKI is induced during EC differentiation downstream of ETS1, resulting in stabilisation of VE‐cadherin expression and modulation of VEGFR transcriptional activation and VEGF secretion, through direct binding to the 3' UTR of STAT3, which leads to its activation. iPS‐ECs overexpressing QKI significantly improved angiogenesis, neovascularization and blood flow recovery in experimental hind limb ischemia. [file STEM-35-952-s011.TIF]
