# Supplementary material for: Quaking Is a Key Regulator of Endothelial Cell Differentiation, Neovascularization, and Angiogenesis
Source: Stem Cells. 2017 Mar 5;35(4):952–66. doi: 10.1002/stem.2594 (PMC5396345; doi:10.1002/stem.2594)
Supplement: Supplementary file 12 — Supporting Information 1 [file STEM-35-952-s012.pdf]

SI Appendix, **Experimental Procedures**

Cochrane, Kelaini, at al.,

Materials

Cell culture media, serum, and cell culture supplements were purchased from ATCC, Millipore, LONZA, and Thermo Fisher Scientific. Antibodies against VE-cadherin (CD144) (ab33168), PECAM (CD31) (ab28364), Flk-1 (ab9530), STAT3 (ab119352), pSTAT3 (ab76315), JAK-1 (ab47435), GAPDH (ab8245), OCT4 (ab19857), eNOS (ab66127), QKI (ab126742) were purchased from Abcam. Antibodies against vWF (SC-8068), QKI (SC-103851) were purchased from Santa Cruz. Antibodies against QKI 5 (AB 9904) were purchased from Millipore. The human specific CD31 antibody was from R&D Systems. SMA-CY3 was purchased from SIGMA. The secondary antibodies for immunostaining anti-mouse Alexa 568, and anti-rabbit Alexa 488, anti-rabbit Alexa 568, anti-goat Alexa 568, anti-goat alexa 488 were purchased from Thermo Fisher Scientific. The secondary antibodies for Western blotting were purchased from Abcam and Cell Signalling. Recombinant mouse VEGF was purchased from Thermo Fisher Scientific. Inhibitors for STAT3 (S1155, working concentration; 250µM) and JAK1/2 (S2219, working concentration 10µM) were from Selleckchem. Actinomycin D (A9415, working concentration; 1 µg/ml), and cycloheximide solution (C4859, working concentration 10µg/ml) were purchased from Sigma-Aldrich.

Mouse iPS and ES cell Culture and Differentiation

Mouse iPS cells were generated in our laboratory using a similar approach as that previously described [1, 2]. Mouse iPS cells were cultured on gelatin-coated flasks (PBS containing 0.04% gelatin from bovine skin; Sigma-Aldrich) in DMEM (ATCC) supplemented with 10% fetal bovine serum, 100 IU/ml penicillin, and 100 µg/ml streptomycin (Thermo Fisher Scientific), 10 ng/ml recombinant human leukemia inhibitory factor (Millipore); and 0.1 mM 2-mercaptoethanol (Invitrogen) in a humidified incubator supplemented with 5% CO_2. The cells

were passaged every 2 days at a ratio of 1:6. Differentiation of iPS cells was induced by seeding the cells on type IV mouse collagen (5 μg/ml)-coated dishes in differentiation medium (DM) containing α-MEM supplemented with 10% FBS (Invitrogen), 0.05 mM 2-mercaptoethanol, 100 units/ml penicillin, and 100 μg/ml streptomycin in the presence of 25 ng/ml VEGF for the time points indicated.

Human iPS cells differentiation and HUVECs

Human iPS cells were pre-differentiated in low attachment plates using StemPro serum free media supplemented with BMP4, Activin A, FGF and VEGF for 5 days. The pre-differentiated cells were seeded on fibronectin (Sigma-Aldrich), whilst KDR endothelial precursor cells were magnetically sorted on day 6 using MicroBeads Kit (Miltenyi BIotec) and culturing in EGM-2 media (LONZA) for 3 to 9 days. QKI was overexpressed or knockdown by lentiviral gene transfer on day 3 after KDR selection and the cells were harvested on day 3 and subjected to further analysis or labeled for the in vivo Matrigel plug assays. HUVECs were seeded on collagen I coated plates and cultured in EGM-2 media. QKI was overexpressed by lentiviral gene transfer and the cells were harvested 48 hours later and subjected to further analysis.

Enzyme-linked Immunosorbent Assay

The concentration of VEGF released in to the supernatant of 6 days differentiated ECs was detected by VEGF ELISA kit (R&D) according to the manufacturer's procedure.

Cell sorting for CD144 positive cells

Cells were tyrpsinized and counted on day 5 of the differentiation towards ECs from iPSCs. $1x10^6$ cells were stained for CD144 and sorted using the Sony SH800 cell sorter. Cells were reseeded on Collagen IV and harvested 5 days later for analysis of EC marker expression and the mRNA level.

Generation of FUW-QKI construct

Primers were designed for QKI (NM_006775) (*5'-CAG CTT GAA TTC ATG GTC GGG GAA ATG GAA-3' and reverse 5'-CGT ATT GGA TCC TTA GTT GCC GGT GGC GGC-3'*) containing restriction enzymes ECOR1 (Thermoscientific #FD0274) with the forward primer and BAMH1 (Thermoscientific #FD0055) with the reverse primer sites. Conventional PCR was preformed using Platinum supermix (Life Technologies #12532-016) following manufactures protocol. PCR product is purified and digested along with FUW vector (a gift from David Baltimore (Addgene plasmid #14882). FUW plasmid underwent additional incubation with alkaline phosphatase (Thermoscientific #EF0654) to stop self-ligation. Two constructs were then ligated overnight (Thermoscientific #K1423). Plasmids were amplified by incubating in Competent Cells (Promega #JM109) for 30 minutes on ice. After a heat shock of 42°C for 90 seconds. Samples were centrifuged for 2 minutes at 4000rpm and only 200µl of the supernatant used to resuspend pellet and spread onto agar plates supplemented with antibiotic resistance. Positive colonies confirmed by restriction enzyme digest and samples sequence.

Generation of KH Domain-QKI mutant construct

Primers were designed either side of the KH Domain of QKI (5'-*TCC CTA ATC ACT GTG GAA GAT-3' and reverse 5'-TCC AGT TTC TCT TGT AAC TGA -3'; 5'TCC* AGC ATC TAA ATG AAG ATT-*3' and reverse 5'-CTT TGC CTC GGA CCA TGA-3'.)* in order to loop out the domain from the pENTR-QKI plasmid (a gift from Huda Zoghbi (Addgene plasmid # 16183)) the linear plasmid ends were then phosphorylated using a polynucleotide kinase (New England Biolabs F7-M0201S). The plasmid then underwent ligation amplification and verification by sequencing as described previously.

Cloning and Nucleofection

The QKI isoforms 5 (217EX-T4215-Lv224) and 6 (217EX-H2552-Lv224) were designed and purchased from Genecopoeia. In addition, we have also cloned these constructs and experiments have been performed in parallel. Full length human QKI 5 and QKI 6 cDNA fragment was obtained by RT-PCR from iPS-ECs with the primer sets: *5'>CAG CAT GGA TCC ATG GTC GGG GAA ATG GAA<3'* and reverse *5'>CGT AGT GAA TTC TTA GTT GCC GGT GGC GGC<3'*, for QKI 5 and *5'>CAG CAT GGA TCC ATG GTC GGG GAA ATG GAA<3'* and reverse *5'>CGC AGC GAA TTC TTA GCC TTT CGT TGG GAA<3'* for QKI 6 and cloned into BamHI and EcoRI sites of a FUW empty plasmid defined as FUW-QKI (5) and FUW-QKI 6. The FUW empty vector was generated from the plasmid FUW-OSKM, (a gift from Rudolf Jaenisch; Addgene plasmid # 20328)[3]. The constructs were verified by DNA sequencing. iPS cells were pre-differentiated for 4 days and then nuclofected with the above plasmids or empty vectors using Amaxa nucleofector (VPH-1001 Kit, LONZA), and cultured in DM+V media for 2 additional days. The differentiated cells were harvested on day 6 and endothelial marker expression was tested at both transcriptional and protein level. The efficiency of the nucleofection was 60-70%. For the early differentiation experiments the QKI constructs have been overexpressed in undifferentiated iPS cells and the samples have been harvested on day 3 and 6 and subjected to further analysis.

Reverse transcriptase-polymerase chain reaction (RT-PCR) and Quantitative RT-PCR

RT-PCR and real time PCR were performed as described previously [4]. Total RNA was extracted using the RNeasy Mini Kit (Qiagen) according to the manufacturer's protocol. 2 µg RNA were reversely transcribed into cDNA with random primer by reverse transcriptase (RT) (Thermo Fisher Scientific). 20-50ng cDNA (relative to RNA amount) was amplified by quantitative RT-PCR. Relative gene expression was determined by quantitative real time PCR, using 2 ng of cDNA (relative to RNA amount) for each sample with the SYBR Green

Master Mix in a 10-µl reaction. *Ct* values were measured using a LightCycler 480 sequence detector (Roche). GAPDH served as the endogenous control to normalize the amounts of RNA in each sample. For each sample, PCR was performed in duplicate in a 384-well reaction plate (LightCycler 480 Roche real time PCR plates). The gene was considered undetectable beyond 35 cycles. The primer sets used for this study are as follows: *VE-cadherin, forward, 5'-AAGAAACCGCTGATCGGCA-3' and reverse, 5'-TCGGAAGAATTGGCCTCTGTC-3'; CD31, forward, 5'-CAAACAGAAACCCGTG GAGAT-3' and reverse, 5'-ACCGTAATGGCTGTTGGCTTC-3'; Flk-1, forward, 5'-TGAAATTGAGCTATCTGCC GG-3' and reverse, 5'-TTTGAAGGTGGAGAGT GCCAG-3'; VegfA, forward, 5'-TCACCAAAGCCAGCACATAGGAGA-3', and reverse, 5'-TTACACGTCTGCGGATCTTGGACA-3'; Stat3, forward 5'-AGTCACATGCCACGT TGGTGTTTC-3', and reverse, 5-’>TCAGTCACAATCAGGGAAGC-3’ Jag-1, forward 5’-CTGTCCCACTGGTTTCTCTG-3’; and reverse, 5’-GTTCTTGCCCTCAT AGTCCTC-3’; Notch 1, forward 5’-ATGTCAATGTTCGAGGACCAG-3’, and reverse, 5’-TCACTGTTGCCT GTCTCAAG-3’; QKI 5, 5’-ATGAATACCCCTACACAT-3’ and reverse, 5’-TTAG TTGCCGGTGGCGGC-3’; QKI 5, forward 5’ CTCTTGCA GCAACAGCCCAGG C-3’ and reverse, 5’ TTAGTTGCCGGTGGCGGCTCGG-3’; QKI 6, 5’-CTCTTGCAGCAACAGCCCAGGC-3’, and reverse 5’-TTAGCCTTTC GTTGGGAAAGCC-3’; QKI 6, 5’-ATGAATACCCCTACACAT-3’ and reverse 5’-CTTAGCCTTTTGGGA-3’; QKI-7,* 5'>CTC TTG CAG CAA CAG CCC AGG C<3' and reverse 5'>TCA ATG GGC TGA AAT ATC AGG C<3'*; GAPDH; 5’-TGTGATGGGTGTGAACCACGAGAA-3’ and reverse 5’-GAGCCCTTCCACAATG CCAAAGTT-3’; eNOS, 5’-TGATGGC GAAGCGAGTGAA-3’ and reverse 5’-ACTCATCCATACACAGGACCCG-3’; AP1, 5’-TGGTTTCCATCTATGGGAGCAGCA-3’ and reverse 5’-TCCATTTGTCTGCACGA TCTCCGA-3’; JAK1, 5’-GCACGGAACCAATGACAACGAACA-3’ and reverse 5’-ACTCCAGTGAACTGGCATCAAGGA-3’; RUNX1; 5’-CCAGGTTGCAAGATTTAATG ACC-3’ and reverse 5’-TTTTGATGGCTCTGTGGTAGG-3’; GATA3 5’-GCGGGCT CTATCACAAAATG-3’ and reverse 5’-TC CCCATTGGCATTCCTC-3’; SF3B1 5’-*

*TCCCAGCCTCCATCAAAAC-3' and reverse 5'-GGTGTCTCATCCCATCTTAAGG-3'; wnt3, 5'-GTGTTAGTGTCCAGGGAGTTC-3' and reverse 5'-CATTTGAGGT GCATGTGGTC-3'; TCF3, 5'-GAGAAGCCCCAGACCAAAC-3' and reverse 5'-ACCACACCTGACACCTTTTC-3'; Hey1, 5'-TGGTAC CCAGTGCTT TTGAG-3' and reverse 5'-CTCCGATAGTCCATAGCAAGG-3';. Notch 4; 5'-AGC TGCCTTGATC TTCCAG-3' and reverse 5'-GCCTTGTCTTTCTGG TCCTTAC-3'. Ets1 5'-*GATCCTGCAGAAAGAGGATGT-3' and reverse 5'-CTCGATACCGTAGCTGATGAAG-3'; *Ephrin B2 (Efnb2) 5'-AGACAAGAGCCATGAAGATCC-3', and reverse 5'-ACTTCTCCCATTTGTACCAGC-3'; CoupTFII 5'-TCAAAGTGGGCATGAGACG -3', and reverse 5'-CAGGTACGAGTGGCAGTTG -3'; Lyve1 5'-CAGCATTCAAGAACGAAGCAG -3', and reverse 5'-GCCTTCACATACCTTTTCACG -3'; Nestin 5'-AAGACTTCCCTCAGCTTTCAG -3'. And reverse 5'-AGCAAAGATCCAAGACGCC -3'; Mef2c 5'-TGTAACACATCGACCTCCAAG -3', and reverse 5'-TGTTCAAGTTACCAGGTGAGAC -3'; STAT3 3' UTR QKI binding site -5'-*CTA CTA ACT TTG TGG TTC CAG-3', and reverse 5'-AGG CAC CAG GAG GCA CTT-3';

ETS1 5'-TCCAGTCCAATTATCACCAGC-3' and reverse 5'-TGCTTGGAGTTAATAGTGGGAC-3';

EPHRIN B2 *5'GAATTCAGCCCTAACCTCTGG-3' and reverse 5'-ATCTTCATGGCTCTTGTCTGG-3'; LYVE1 5'- TTAGCCCAAACCCCAAGTG-3' and reverse 5'- TCTGGAATGCACGAGTTAGTC -3'.*

Immunofluorescence staining

The procedure used for immunofluorescent staining was similar to that described previously [4]. Briefly, cells were fixed with 4% paraformaldehyde and permeabilised with 0.1% Triton X-100 in PBS for 10 min and blocked in 5% swine or donkey serum in PBS for 30 min at 37˚C. The cells were incubated with primary antibody: rabbit CD144, mouse VEGFR (Flk-1), rabbit eNOS, rabbit CD31, goat vWF, goat or rabbit QKI for 1 h at 37˚C. The bound primary

antibody was revealed by incubation with the secondary antibody; anti-mouse Alexa 568, and anti-rabbit Alexa 488, anti-rabbit Alexa 568, anti-goat Alexa 568, anti-goat alexa 488 at 37°C for 45 min. Cells were counterstained with 4',6-diamidino-2-phenylindole (DAPI; Sigma-Aldrich), mounted in Floromount-G (Cytomation; DAKO, Glostrup, Denmark), and examined with a fluorescence microscope (Axioplan 2 imaging; Zeiss) or SP5 confocal microscope (Leica, Germany).

Immunoblotting

The method used was similar to that described previously [4]. Cells were harvested and washed with cold PBS, re-suspended in lysis buffer (25mM Tris-Cl pH 7.5, 120mM NaCl, 1 mM EDTA pH 8.0, 0.5% Triton X100) supplemented with protease inhibitors (Roche) and lysed by ultra-sonication (twice, 6 seconds each) (Bradson Sonifier150) to obtain whole cell lysate. The protein concentration was determined using the Biorad Protein Assay Reagent. 50µg of whole lysate was applied to SDS-PAGE and transferred to Hybond PVDF membrane (GE Health), followed by standard western blot procedure. The bound primary antibodies were detected by the use of horseradish peroxidase (HRP)-conjugated secondary antibody and the ECL detection system (GE Health). The band density was semiquantified by Adobe Photoshop software.

Luciferase Reporter Assay

For the luciferase reporter assays, 3×10^4 iPS cells were seeded on collagen-coated well of a 12-well plate in DM+V. 72 h later, cells were transfected with the FUW-QKI (5), and control plasmids under the control of the VEGFR promoter (pGL3-VEGFR2-780 was a gift from Donald Ingber Addgene plasmid # 21307)[5], or the PGL3-Luc-CD144 [4], or the 3' UTR of STAT3 (CS-HmiT017767-MT06), or the 3' UTR of STAT3 where the QKI Motif was deleted (CS-HmiT017767-MT06-01 (custom made constructs from Genecopoeia) Briefly, 0.33 µg/well of the reporter plasmids were co-transfected with the FUW-QKI and controls (0.17

μg/well) using FUGENE 6 according to the protocol provided. pGL3-Luc Renilla (0.1μg/well) was included in all transfection assays as an internal control. Luciferase and Renilla (Promega) activity assays were detected 48hr after transfection using a standard protocol[6]. Relative luciferase units (RLU) were defined as the ratio of luciferase activity to Renilla activity with that of control set as 1.0.

RNA Binding Protein Immunoprecipitation Assays

RNA Binding Protein Immunoprecipitation Assays were performed on 3 days differentiated iPS-ECs using the Magna RIP kit from Millipore (17-700) according to the protocol provided. A QKI-5 specific antibody from Millipore and rabbit IgG purified antibodies (provided in the kit) have been used. The purified RNA was subjected to RT-PCR using specific primers for the binding site of QKI motif in 3'UTR region of STAT3.

Lentiviral particle transduction

Lentiviral particles were produced using MISSION shSTAT3, shQKI plasmids DNA (Sigma-Aldrich) according to the protocol provided and as previously described[4]. The shRNA Non-Targeting vector was used as a negative control. Briefly, 293T cells were transfected with the lentiviral vector and the packaging plasmids, pCMV-dR8.2 and pCMV-VSV-G (both obtained from Addgene) using Fugene 6. The supernatant containing the lentivirus was harvested 48h later, filtered, aliquoted and stored at –80°C. p24 antigen ELISA (Zeptometrix) was used to determine the viral titre. The Transducing Unit (TU) was calculated using the conversion factor recommended by the manufacturer (10^4 physical particles per pg of p24 and 1 transducing unit per 10^3 physical particles for a VSV-G pseudotyped lentiviral vector), with 1pg of p24 antigen converted to 10 Transducing Units (TU). For the lentiviral particle transduction for the constructs purchased from Genecopoeia, a similar approach was used following the manufacturer's instructions. For lentiviral infection, iPS cells were differentiated

for 3-4 days and the cells were incubated with shSTAT3, or shQKI or non-targeting control ($1x10^7$TU/ml), or QKI 5 (217EX-T4215-Lv224) and QKI 6 (217EX-H2552-Lv224) in complete medium supplemented with 10μg/ml of Polybrene for 24h. Subsequently, fresh medium was added to the cells and the plates were returned to the incubator and harvested 48h -72h later to be subjected to further analysis. The efficiency of the infection in iPS cells during EC differentiation was 70-80%.

In vitro tube formation assay

iPS cells were differentiated in the presence or absence of VEGF for 4 days and then transfected or infected with the QKI or empty vector constructs. *In vitro* angiogenesis assays were performed after 48 h as described previously[1, 6]. Cell suspension containing 4×10^4 transfected cells were placed on top of the 50 μl/well Matrigel (10 mg/ml; BD Matrigel Basement Membrane Matrix, A6661) in 8-well chamber slides (Nunc). Rearrangement of cells and the formation of capillary-like structures were observed at 6-12 h.

In vivo Matrigel Plug assay

In vivo angiogenesis was assessed 48 h after QKI infection by mixing 5×10^5 mouse iPS cells with 50 μl of Matrigel prior to subcutaneous injection in to the flank regions of male C57BL/6 mice. Human iPS-ECs control cells or iPS-QKI-ECs were labelled with Molecular Probes Vybrant Cell Labelling (MP22885) before the in vivo angiogenesis assay to distinguish the in vitro–differentiated cells from the host cells. The cells ($1x10^6$) were mixed with 50 μl of Matrigel and injected subcutaneously into the back or flank of NOD.CB17-Prkdcscid/NcrCrl mice. Six injections were conducted for each group. Seven days later, the mice were killed and the plugs were harvested, frozen in liquid nitrogen, and cryosectioned. Samples were fixed with 4% paraformaldehyde in PBS at 4°C overnight, and then HE staining was performed at room temperature. Images were assessed with Axioplan 2

imaging microscope with Plan-NEOFLUAR 10×, NA 0.3, objective lenses, AxioCam camera, and Axiovision software (all Carl Zeiss MicroImaging, Inc.) All procedures were performed in accordance with the Guidance on the Operation of the Animals (Scientific Procedures) Act, 1986 (UK) and were approved by the Queen's University Belfast Animal Welfare and Ethical Review Body.

Immunofluoresence staining and frozen section staining

The procedure for frozen section staining was similar to that described elsewhere [7]. In short, Matrigel plugs were harvested, serial 5 mm-thick frozen sections were cut from cryopreserved tissue blocks, fixed in cold 1:1 acetone for 10 min, and washed with phosphate-buffered saline (PBS) for 20 min. Specimens were then placed in a humidified chamber and blocked in 5% swine serum in PBS for 30 min at 37˚C and incubated with primary antibodies rabbit anti-VE-cadherin and rabbit anti-CD31, prior to immunostaining, as described above. The bound primary antibodies were revealed by incubation with the secondary antibody; anti-rabbit Alexa488, at 37°C for 45 min. Specimens were counterstained with 4',6-diamidino-2-phenylindole (DAPI; Sigma-Aldrich), mounted in Floromount-G (Cytomation; DAKO, Glostrup, Denmark), and examined with a fluorescence microscope (Axioplan 2 imaging; Zeiss) or SP5 confocal microscope (Leica, Germany). Immunostaining was assessed and capillary density was calculated as capillary number/mm^2.

Experimental hind limb ischemia

The mouse hind limb ischemia model was performed as previously described [6, 8]. iPS-ECs were infected with FUW-QKI or control empty vectors on day 4 of EC differentiation. 48 h later the cells were trypsinised and labelled with Molecular Probes Vybrant Cell Labelling (MP22885) before being injected intramuscularly into the adductors of ischemic C57 wild

type mice. Tissue blood flow of both legs was sequentially assessed by Laser Doppler imaging (moorLDI2-IR). Seven and 14 days later, mice were sacrificed, and hind limb muscles were harvested following in situ perfusion fixation at physiological pressure, frozen in liquid nitrogen, and cryosectioned for assessment of neoangiogenesis. Sections of adductor muscles were stained with CD144 and CD31 antibody and capillary density was expressed as capillary number per mm^2. Cell engraftment ability was assessed by counting cells double positive for Vybrant and EC markers (CD144 or CD31) at six randomly selected microscopic fields (at x40).

Statistical Analysis

Data are expressed as the mean±SEM and were analyzed using GraphPad Prism 5 software with a two-tailed Student's *t* test for two groups or pairwise comparisons or ANOVA. A value of *: $p<0.05$. * $p<0.05$, ** $p<0.01$, *** $p<0.001$ was considered significant.

References

1. Di Bernardini, E. *et al.* Endothelial lineage differentiation from induced pluripotent stem cells is regulated by microRNA-21 and transforming growth factor beta2 (TGF-beta2) pathways. *J Biol Chem* **289**, 3383-3393 (2014).
2. Galarneau, A. & Richard, S. Target RNA motif and target mRNAs of the Quaking STAR protein. *Nature structural & molecular biology* **12**, 691-698 (2005).
3. Carey, B.W. *et al.* Reprogramming of murine and human somatic cells using a single polycistronic vector. *Proc Natl Acad Sci U S A* **106**, 157-162 (2009).
4. Margariti, A. *et al.* Histone deacetylase 7 controls endothelial cell growth through modulation of beta-catenin. *Circ Res* **106**, 1202-1211 (2010).
5. Mammoto, A. *et al.* A mechanosensitive transcriptional mechanism that controls angiogenesis. *Nature* **457**, 1103-1108 (2009).
6. Margariti, A. *et al.* Direct reprogramming of fibroblasts into endothelial cells capable of angiogenesis and reendothelialization in tissue-engineered vessels. *Proc Natl Acad Sci U S A* **109**, 13793-13798 (2012).
7. Zeng, L. *et al.* Sustained activation of XBP1 splicing leads to endothelial apoptosis and atherosclerosis development in response to disturbed flow. *Proc Natl Acad Sci U S A* **106**, 8326-8331 (2009).
8. Emanueli, C. *et al.* Nitropravastatin stimulates reparative neovascularisation and improves recovery from limb Ischaemia in type-1 diabetic mice. *Br J Pharmacol* **150**, 873-882 (2007).
